# Super enhancer-driven LINC01013 mediates hypoxia-induced mitochondrial dysfunction by HSPA9 to determine pulmonary arterial smooth muscle cell fate

**DOI:** 10.1007/s00018-025-06071-3

**Published:** 2026-01-06

**Authors:** Cui Ma, Zhaosi Wang, Xiangrui Zhu, Xiangming Pang, Lixin Zhang, Langlin Ou, Yingli Chen, Yuxiang Liu, Jian Mei, Xiaoyu Guan, Zitong Meng, Yujing Tang, Zeying Zhang, Baolei Li, Shiqng Wen, Ao Shen, Xiaoying Wang

**Affiliations:** 1 Institute of Cardiovascular Diseases, Xiamen Cardiovascular Hospital, School of Medicine, Fujian Branch of National Clinical Research Center for Cardiovascular Diseases, Xiamen University, Xiamen, 361006 P. R. China; 2 College of Pharmacy, Harbin Medical University-Daqing Campus, Daqing, 163319 P. R. China; 3 College of Medical Laboratory Science and Technology, Harbin Medical University-Daqing Campus, Daqing, 163319 P. R. China; 4 School of Medical Laboratory, North Henan Medical University, Xinxiang, 453500 P. R. China; 5 College of Pharmacy, Harbin Medical University, Harbin, 150081 P. R. China; 6 College of Pharmacy, Harbin Medical University, Daqing Campus, 39 Xinyang Road Daqing, Heilongjiang, 163319 P. R. China

**Keywords:** Pulmonary hypertension, LncRNA, VDAC1, Proliferation and inflammation

## Abstract

Super-enhancers (SEs) typically govern the expression of critical genes in the maintenance of cell identity. Recent advances suggest mitochondrial dysfunction contributes to pulmonary artery smooth muscle cell (PASMC) proliferation and inflammation in pulmonary hypertension (PH). However, the landscape of SEs in hypoxic PASMCs as well as hypoxia-induced target genes associated with SEs controlling the mitochondrial dysfunction remain to be fully characterized. In this study, we depicted the landscape of SE in hypoxic PASMCs by ChIP-seq, Hi-ChIP, and ChIP-qPCR assays and reveal a regulatory SE driven LncRNA, LINC01013. The effect of LINC01013 on proliferation and inflammation of PASMCs was evaluated through EdU incorporation, Western blotting and immunofluorescence. The molecular mechanism of LINC01013 was investigated by the study of RNA pull down and mass spectrometry. We profiled chromosome interactions in epigenetic regulation and identified SE-associated LINC01013 as a key mitochondrial dysfunction mediator in hypoxic PASMCs. The transcription factor CCAAT enhancer binding protein beta (CEBPB) was found to enrichment in LINC01013 SE and promoter, promoting LINC01013 transcription and overexpression in PASMCs under hypoxic conditions. Inhibition of LINC01013 reversed hypoxia-induced glycolysis and oxidative stress injury of PASMCs. Further investigation unveiled that LINC01013, which is partially located in mitochondria and interacted with heat shock protein family A member 9 (HSPA9) to mediate oligomerization of voltage dependent anion channel 1 (VDAC1), thereby leading to increased mitochondrial permeability and dysfunction. These findings demonstrate that SE-associated LINC01013 regulates the proliferation and inflammation of hypoxic PASMCs by orchestrating mitochondrial function, might be a potential therapeutic target for PH.

**Supplementary Information:**

The online version contains supplementary material available at 10.1007/s00018-025-06071-3.

## Introduction

Pulmonary hypertension (PH) is a cardiopulmonary dysfunction disease, characterized by pulmonary vascular remodeling, elevated pulmonary arteries pressure, and eventually right heart failure and death [1, 2]. Pulmonary artery smooth muscle cell (PASMC) dysfunction is considered as main cause of pulmonary vasculature pathological changes in PH, including proliferation, pyroptosis, autophagy, ferroptosis, and calcification [3–7]. Although, researches conducted over the last several years has highlighted that PASMC hyperproliferation as a hallmark feature in contributing pulmonary vascular remodeling, however, the key regulators that orchestrate and instruct proliferative program in PH are still largely unknown.

Mitochondria are highly dynamic organelles that play vital regulatory roles in oxidative stress and energy metabolism. Accumulating evidence demonstrates that mitochondrial dysfunction plays an essential role in pulmonary vascular remodeling by contributing to the hyperproliferation and inflammation of PASMCs [8]. For instance, Maresin-1 (MaR1) suppressed hypoxia-induced PASMC proliferation and alleviated pulmonary vascular remodeling by improving mitochondrial homeostasis through lipoxin A4 receptor (ALXR)/heat shock protein 90α (HSP90α) axis [9]. Calcitonin gene-related peptide (CGRP) attenuated vascular remodeling by inhibiting the cGAS-STING-NFκB inflammation pathway mediated by injured mitochondria [10]. In addition, our previous studies have reported that miR-125a and apoptosis inducing factor (AIF), which regulate the mitochondrial oxidative phosphorylation and glycolysis, play an important role in PASMC proliferation under hypoxic conditions [11, 12]. Despite the above reports, the mechanisms underlying mitochondrial dysfunction that links to pulmonary vascular lesion in PH are still warrant in-depth investigate.

Long noncoding RNAs (lncRNAs) are transcripts longer than 200 nucleotides and are important versatile molecules involved in pathological process of PH via interactions with DNA, RNA, or proteins. In PASMCs, lncRNA CASC2 can sponge miR-222 and inhibit hypoxia-induced cellular excessive proliferation and migration [13]. Similarly, lncRNA Rps4l induced PASMC cell cycle progression by stabilization of ILF3 (interleukin enhancer-binding factor 3) [14]. In pulmonary artery endothelial cells, lncRNA FENDRR regulates cell pyroptosis by forming RNA-DNA triplexes with dynamin-related protein 1 (DRP1) promoter [15]. However, the current knowledge about the specific lncRNAs involved in mitochondrial dysfunction are still rather limited.

Super enhancers (SEs) drive higher levels of transcription by binding transcription factors, chromatin regulators and cofactors, and regulate cell fate and differentiation [16]. SEs have been identified to promote the occurrence and development of multiple diseases, including polycystic kidney disease, cardiovascular disease, and especially cancers [17–19]. SE-lncRNA refers to lncRNA either interact with SEs or transcript from SEs locus. Interestingly, Tao et al. demonstrated that SE-lncRNA LINC01089 promotes hepatocellular carcinoma metastasis by regulating DIAPH3 alternative splicing [20]. Zhang et al. showed that the SE-lncRNA Snhg7 may regulate cardiac hypertrophy via Tbx5/GLS2/ferroptosis pathway [21]. Therefore, it is very interesting and meaningful to study the role of SE-lncRNA in mitochondrial dysfunction. This new area of study will provide new mechanistic leads for the selective targeting of SE-lncRNA involved in pathological process of PH.

## Materials and methods

### Experimental animals

Adult male C57BL/6 mice (6–9 weeks old, weighing 20–25 g) were used in this study. All experimental procedures were approved by the Institutional Animal Care and Use Committee (IACUC). Clonal constructs of LINC01013 targeting the smooth muscle cell-specific promoter SM22α (smooth muscle 22α) were packaged into serotype 5 adeno-associated virus (AAV5) vectors and synthesized by GeneChem (Shanghai, China) (Fig. S1A). A total of 10¹¹ genome equivalents of smooth muscle cell-specific AAV5-LINC01013 (AAV5-LINC01013^▲SMC^) or AAV5 negative control (AAV5-NC^▲SMC^) were prepared in 20 µL of HBSS. The viruses were administered intranasally to isoflurane-anesthetized mice. 7–14 days post-infection, the mice were randomly allocated to either normoxic or hypoxic groups. To establish the SU5416 and hypoxia (SuHx)-mediated PH model, mice were administered the VEGFR inhibitor SU5416 (20 mg/kg; HY-10374, MCE, USA) via intraperitoneal injection once per week and concurrently exposed to chronic hypoxia (FiO₂ 0.10) for 3 weeks. Control mice were administered the vehicle and maintained under normoxic conditions (FiO₂ 0.21).

### Echocardiography, RV systolic pressure and morphometric analysis

Pulmonary hemodynamic index and cardiac function were evaluated by echocardiography using a Vevo2100 imaging system equipped with an 18- to 38-MHz (MS400, mouse cardiovascular) transducer probe (VisualSonics, Inc, Toronto, Ontario, Canada) in anesthetize mice, as previously described [22]. The RV systolic pressure was monitored using Power Labmonitoring equipment (AD Instruments, Colorado Springs, CO). A 1.2 French Pressure Catheter (Scisense Inc) was surgically inserted into the right jugular vein, and the RV systolic pressure was continuously recorded for 20 to 40 min. Above experiments were performed by the same experienced investigator, and data were analyzed by investigators blinded to treatment. Finally, the heart and lung tissues were removed, the right ventricle was slowly separated along the ventricular septum, and the degree of right heart hypertrophy was calculated.

After measurements in vivo, lung tissues were collected and fixed in 4% paraformaldehyde, embedded in paraffin, and sectioned at 5-µm intervals. Hematoxylin and eosin staining (HE staining) and Masson trichrome staining were performed according to the manufacturer’s protocol. All the analyses were performed by another person who was blinded to the experimental protocol.

### Cell culture and treatment

The human PASMCs were provided by Procell Life Science & Technology (Wuhan, China), placed in smooth muscle cell medium (SMC, Sciencell, 1101, CA, USA) containing 15% fetal bovine serum and 1% penicillin streptomycin, cultured in a 37 °C, 5% CO_2_, and 100% relative humidity. For the hypoxia exposure experiments, cells were placed in a Tri-Gas Incubator (Thermo Fisher, MA, USA) in a water-saturated atmosphere containing 3% O_2_ and 5% CO_2_ for required time.

### ChIP-seq and Hi-Chip

ChIP-seq was performed by Guangzhou Epibiotek Co., Ltd. Briefly, the cells were cross-linked with 1% formaldehyde at room temperature for 10 min, and were quenched with 0.125 M Glycine for 5 min. Cells were lysed in Nuclear Lysis Buffer and followed by sonication to generate 200 to 500 bp fragments. 5 µg of anti-H3K27ac antibody (Cell Signaling) was incubated overnight at 4 ℃ with the fragmented chromatin, and then incubated with protein A/G magnetic beads. ChIP complexes were eluted in reverse cross-linking buffer followed by incubate the tube at 65 ℃. ChIP DNA was used to prepare multiplexed sequencing libraries by using the QIAseq Ultralow Input Library Kit (QIAGEN) according to the manufacturer’s protocol. After library construction, the samples were subjected to sequencing analysis.

For Hi-ChIP, cross-linked cells were lysed in cold Hi-C lysis buffer and followed by Hi-ChIP-restriction enzyme digestion at 37 ℃ for 16 h, fill-in of overhangs with biotin-dATP (Thermo Fisher) at 37 ℃ for 1 h, and ligation with Intra-Aggregate Ligation buffer at room temperature for 4 h. After proximity ligation, the nuclei with in situ generated contacts were pelleted for 5 min at room temperature. 5 µg of anti-H3K27ac antibody (Cell Signaling) was incubated overnight at 4 ℃ with the fragmented chromatin, and then incubated with protein A/G magnetic beads. Biotin labeled post-ChIP DNA was used to prepare multiplexed sequencing libraries according to the manufacturer’s protocol. After library construction, the samples were subjected to sequencing analysis.

### Fluorescent in situ hybridization (FISH)

In order to identify the expression and location of LINC01013, Cy3-labeled LINC01013 probes were synthesized by GenePharma (Shanghai, China). The slide with appropriate numbers of PASMCs was fixed in 4% paraformaldehyde, and then permeabilized with 0.3% Triton X-100. Cy3-labeled LINC01013 probes were added to the hybridization solution, and the cells were incubated in a 37 ℃ incubator overnight. Cells were stained with DAPI staining solution, and captured with a live cell workstation (AF6000; Leica, Wetzlar, Germany). 18 S and U6 probes were used as internal controls.

### Western blotting analysis

The total protein was extracted from the lung tissue or PASMCs with RIPA buffer supplemented with PMSF (P0013B and ST506; Beyotime, Shanghai, China). The protein samples were fractionated by 10%-12% SDS‒polyacrylamide gel and transferred onto nitrocellulose membranes. The antibody against CEBPB (PB9171, BA0670, 1:500, Boster, Wuhan, China), H3K27ac (A7253, 1:500, ABclonal, Wuhan, China), H3K4me1 (A2355, 1:500, Wuhan, China), PCNA (A00125, 1:500, Boster, Wuhan, China), Cyclin A (PB0515, 1:500, Boster, Wuhan, China), Cyclin D (BM4272, 1:500, Boster, Wuhan, China), IL-6 (AF7236, 1:500, Beyotime, Shanghai, China), TNF-α (AF8208, 1:500, Beyotime, Shanghai, China), PKM2 (4053, 1:1000, Cell Signaling, MA, US), HK II (66974-1-Ig, 1:1000, Proteintech, IL, USA), PDH (2784, 1:1000, Cell Signaling, MA, US), HSPA9 (14887-1-AP, 1:5000, Proteintech, IL, USA), VDAC1 (10866-1-AP, 1:5000, Proteintech, IL, USA), and β-actin (TA-09, 1:1000, ZSGB‑BIO, Beijing, China) was incubated at 4 °C overnight, followed by incubation with appropriate horseradish peroxidase-conjugated secondary antibodies at room temperature for 1 h, and proteins were visualized with enhanced chemiluminescence reagents.

### Real-time RT-PCR (RT-qPCR)

The total RNA of tissue or PASMCs samples was extracted by TRIzol reagent (Thermo Fisher, MA) according to the manufacturer’s protocol. Cytoplasmic and nuclear RNAs were isolated and purified using Norgen’s Cytoplasmic & Nuclear RNA Purification Kit (Thorold, ON, Canada). For each sample, the extracted RNA was reverse transcribed to cDNA using the Superscript First-Strand Complementary DNA Synthesis Kit (HaiGene, Harbin, China). Quantitative real-time PCR was performed using SYBR Green (TOYOBO, Osaka, Japan) on LightCycler 480 II real-time PCR system (Roche, Basel, Switzerland). β-actin, 18s or U6 were used as internal controls. The threshold cycle (Ct) was determined, and the data were calculated by the relative quantitative 2^−ΔΔCT^ method. The sequences of the primers used are listed in Supplementary Table S1.

### 5-ethynyl-2’-deoxyuridine (EdU) incorporation assay

EdU assays were performed by using the BeyoClick EdU cell proliferation kit according to the manufacturer’s instructions (C0075S, Beyotime, Shanghai, China). The slide with appropriate numbers of PASMCs was incubated with EdU for 2 h at 37 °C, and fluorescence at a wavelength of 555 nm was detected by live cell workstation (AF6000; Leica, Wetzlar, Germany).

### Glycolysis assay

PASMCs were seeded in 24-well multiwell plates (102340-100; Agilent, CA) and detect the extracellular acidification rate (ECAR, indicative of glycolysis) was detected by using a Seahorse XF24 Extracellular Flux Analyzer (Agilent Technologies Co, Ltd). On the day of the measurement, the cells were incubated at 37 ℃ for 1 h in the CO_2_ free incubator to balance the media pH and temperature, and then treated with glucose (2 mg/mL), oligomycin (1 µM), and 2-deoxy-D-glucose (100 mmol/L). Results were normalized to total cell count of each well.

### Chromatin immunoprecipitation–quantitative PCR (ChIP–qPCR)

ChIP-qPCR assay was performed to determine the enrichment of CEBPB, H3K27ac and H3K4me1 in the SE and promoter region of LINC01013 in PASMCs by using a ChIP Assay Kits (P2078, Beyotime, Shanghai, China) according to the manufacturer’s protocol. Briefly, PASMCs were cross-linked with 1% formaldehyde, followed by ultrasonic shear to generate 200–1000 bp size fragments of the genomic DNA. Antibodies against H3K27ac (A7253, 1:100, ABclonal, Wuhan, China), H3K4me1 (A2355, 1:100, ABclonal, Wuhan, China), CEBPB (18311-1-AP, 1:150, Proteintech, IL, USA) and normal rabbit IgG were used for immunoprecipitation. The purified DNA was finally examined by RT-qPCR. The sequences of the primers used are listed in Supplementary Table S1.

### RNA binding protein immunoprecipitation (RIP) and protein coimmunoprecipitation (Co-IP) assays

RIP assays were performed according to the manufacturer’s protocol (Bes5101, BersinBio, Guangzhou, China). Briefly, the cells were collected and lysed in RIP lysis buffer, followed by incubated with 20 µL of protein A/G bead-conjugated anti-HSPA9 antibody (5 µL, 16018-1-AP; Proteintech, IL, USA) and rabbit IgG antibody (5 µL, A6053; ABclonal, Wuhan, China). The precipitated RNA samples were converted to cDNA and subjected to RT-qPCR assays. For the protein coimmunoprecipitation assay, cells were lysed in lysis buffer (Tris 50 mM, pH 7.4; NaCl 150 mM; Triton X-100 1%; EDTA 1 mM; and PMSF 2 mM) and then incubated with 5 µg of the anti-H3K27ac, anti-H3K4me1, anti-CEBPB antibodies and protein A + G agarose beads overnight at 4 °C. The antibody-protein complexes were examined using Western blotting.

### RNA Pull-down and mass spectrum assays

RNA pull-down assay was performed to determine the binding of LINC01013 and HSPA9 proteins according to the manufacturer’s protocol (Bes5102, BersinBio, Guangzhou, China). The biotinylated LINC01013 probe was synthesized by GenePharma (Shanghai, China). Cells were lysed and incubated with magnetic beads coupled with the LINC01013 probe or a negative control (NC) probe for 2 h at 25 °C. The recovered proteins were then examined by using Western blotting, silver staining analysis and mass spectrometry analysis. The mass spectrometry analysis was performed by Beijing Bio-Tech Pack Technology Company Ltd.

### Cell and tissue immunofluorescence staining analysis

Prepared cells were fixed with 4% paraformaldehyde, permeabilized with 0.3% Triton X-100, and blocked with 5% bovine serum albumin. Then, the cells were incubated with antibodies against HSPA9 (14887-1-AP, 1:100, Proteintech, IL, USA) at 4 °C overnight. After washing three times with cold PBS, the cells were incubated with Cy3/FITC conjugated secondary antibody (1:100) at 37 °C for 2 h. Nuclei were stained with DAPI before the results were observed. Frozen sectioning of mouse lung tissues was performed in the same manner with TNF-α (AF8208, 1:100, Beyotime, Shanghai, China).

### Superoxide dismutase and glutathione peroxidase activity assay

According to the manufacturer’s protocol, SOD (superoxide dismutase) and GPx (glutathione peroxidase) activity were measured by using colorimetric CuZn/Mn-SOD activity assay kit and GPx activity assay kit (S0103 and S0058, Beyotime, Shanghai, China), respectively.

### Measurement of mitochondrial ROS and mitochondrial membrane potential

Mitochondrial ROS and mitochondrial membrane potential were measured after cells incubation with MitoSOX Red (M36008; Thermo Fisher, MA) or JC-1 (C2006, Beyotime, Shanghai, China) for 30 min at 37 °C, and fluorescence was captured (470 nm excitation/530 nm emission) from ≥ 3 optical fields. The fluorescence intensity was quantified by Image J.

### Cross-linking experiments

Cells were harvested after treatment and were incubated with cross-linking reagents 100 and 500 µM Ethylene glycolbis (EGS) for 30 min at 30 °C. Samples were subjected to SDS-PAGE and immunoblotting using VDAC1 specific antibodies, and immunoreactive VDAC dimer and trimer bands were quantitated respectively.

### Plasmid and siRNA construction and transfection

Cell transfection experiments were performed according to the manufacturer’s instructions for the transfection reagent (X-tremeGene siRNA or Lipofectamine 2000), and the procedure for transfection was described previously [23]. An overexpression vector for HSPA9 was constructed using the GV658 vector, and an empty vector alone was used as a negative control (Genechem, Shanghai, China). Small interfering RNA (siRNA) against LINC0103, and nontargeted control siRNA were designed and synthesized by GenePharma (Shanghai, China). The sequences are listed in Supplementary Table S2.

### Statistical analysis

The results are expressed as means ± SEM. Statistical analyses were conducted using GraphPad Prism 8 (GraphPad, La Jolla, CA, USA). All data were tested for normal distribution using the Shapiro-Wilk test and equal variance (F test), with *P* ≤ 0.05 was considered statistically significant. Statistical analysis and calculation of the *P* values were performed by unpaired 2-tailed Student t test (normally distributed with equal variance), the Welch correction test (normally distributed with unequal variance) or Mann-Whitney U test (non-normally distributed) for comparing 2 experimental groups and one-way ANOVA with Tukey post hoc test (normally distributed with equal variance), Brown Forsythe and Welch ANOVA with Tamhane T2 post hoc test (normally distributed with unequal variance) or Kruskal-Wallis test followed by the Dunn post-test (non-normally distributed) for comparing ≥ 3 experimental groups.

## Results

### Identification of LINC01013 as a SE-driven LncRNA associated with hypoxia

**Fig. 1:**
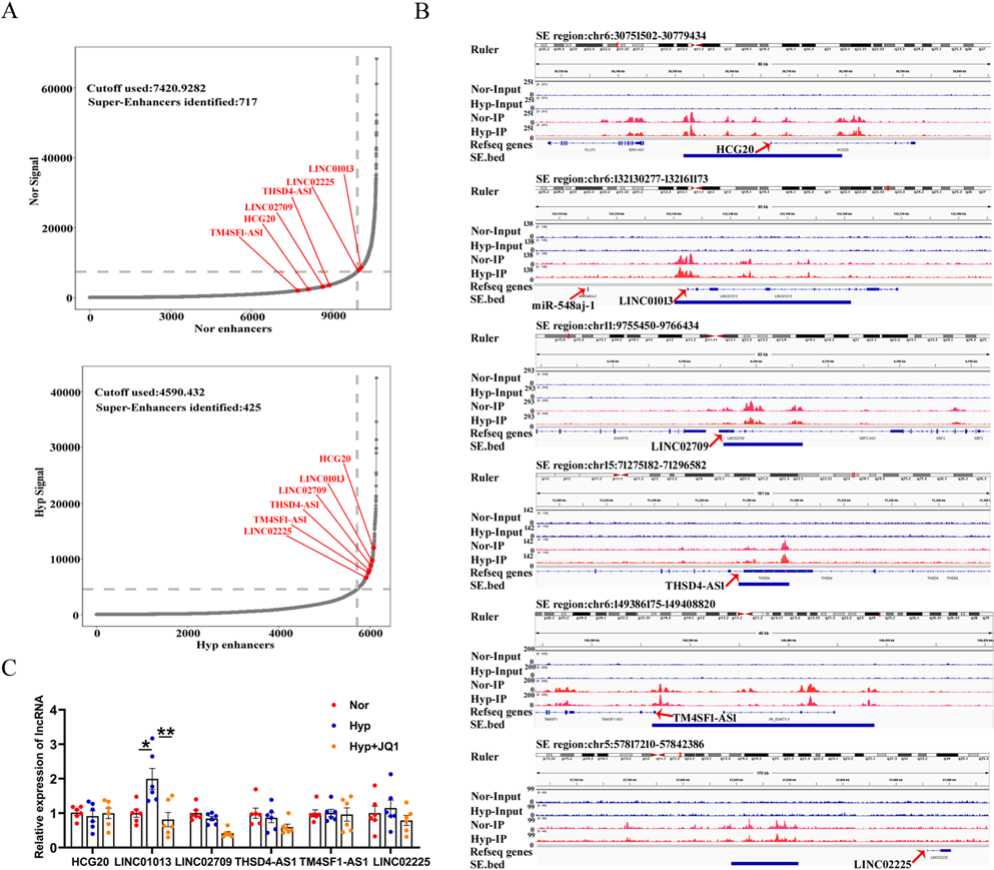
LINC01013 was a SE-driven gene in hypoxia. **A** The hockey stick plot illustrates the activity of the top 6 SE–associated lncRNAs in hypoxic PASMCs—where SEs are defined by a slope > 1, and typical enhancers by a slope < 1—based on chromatin immunoprecipitation sequencing (ChIP-seq) using an antibody targeting H3K27ac. **B** Gene tracks depicting the SE region of HCG20, LINC01013, LINC02709, THSD4-AS1, TM4SF1-AS1 and LINC02225 in hPASMCs by Integrative Genome Viewer. MiR-548aj-1 is the gene closest to LINC01013. **C** We quantified the expression of SE-associated lncRNAs using RT-qPCR in hypoxic hPASMCs treated with JQ1, with β-actin used as a internal reference gene (*n* = 6). All values are presented as the mean ± SEM. Statistical analysis was performed with one-way ANOVA. *p < 0.05, **p < 0.01. Nor, normoxia; Hyp, hypoxia

**Fig. 2:**
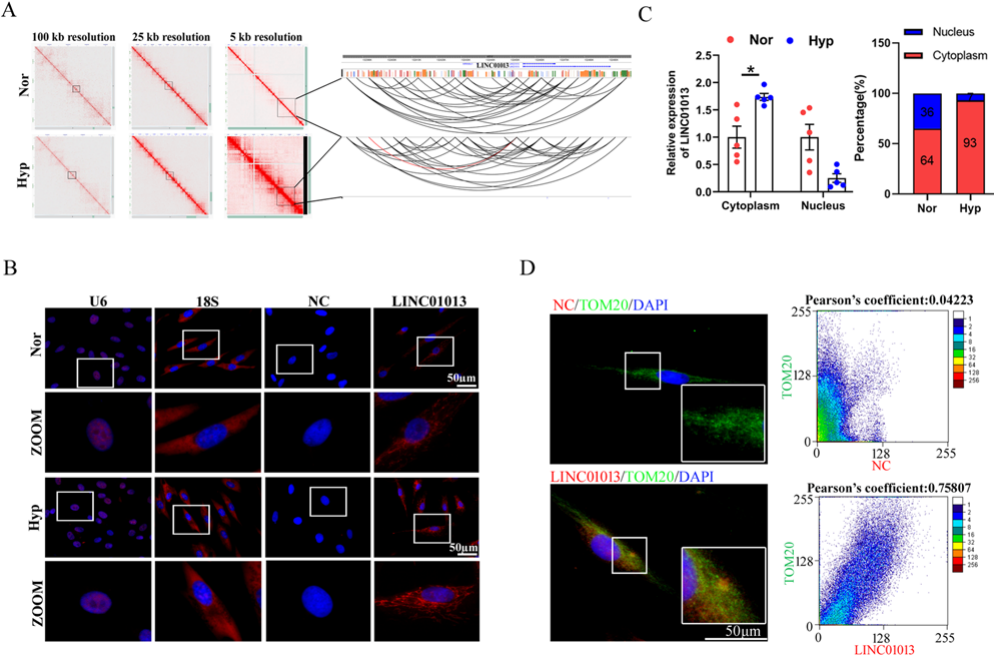
The subcellular localization of LINC01013.** A** HiChIP data displaying spatial interactions between the SE and promoter of LINC01013 under nomorxic and hypoxic conditions. **B** RNA-FISH was performed to determine the LINC01013 distribution and expression in hPASMCs. 18 S and U6 were used as cytoplasmic and nuclear marker, respectively Scale bar, 50 μm. **C** Cytoplasmic and nuclear RNA separation followed by RT-qPCR assay. 18 S and U6 were used as cytoplasmic and nuclear internal reference gene, respectively (*n* = 5). **D** RNA-FISH analysis demonstrated the partial mitochondrial localization of LINC01013 in hPASMCs, using TOM20 as a mitochondrial marker and a non-targeting FISH probe as a negative control (NC). All values are presented as the mean ± SEM. Statistical analysis was performed with Student’s t-test. *p < 0.05. Nor, normoxia; Hyp, hypoxia; NC, negative control

To explore SE-associated lncRNAs involved in the pathological process of PH, we profiled epigenomics differences by perform ChIP-seq assay with antibodies against extensive active histone mark, histone 3 lysine 27 acetylation (H3K27ac) in hypoxic human pulmonary artery smooth muscle cells (hPASMCs). We identified the top 6 SE-lncRNAs that exhibited significant H3K27ac enrichment under hypoxic conditions, including HCG20, LINC01013, LINC02709, THSD4-AS1, TM4SF1-AS1, and LINC02225 (Fig. 1A). We then visualized H3K27ac peaks near the genomic loci of these genes in both hypoxic and normoxic conditions using the Integrative Genome Viewer (Fig. 1B). A up-regulation of LINC01013 (AERRIE; Transcript NR_038981.2) was observed in hypoxic compared to normoxic cells. Notably, this induction was suppressed by JQ-1, an inhibitor of BRD4—a key regulator of SEs (Fig. 1C). Furthermore, to investigate whether the proposed SE region of LINC01013 influences other adjacent genes, we examined miR-548aj-1 (as indicated in Fig. 1B) and analyzed its expression under hypoxic conditions using RT-qPCR. The results revealed that the expression of miR-548aj-1 was down-regulated under hypoxia, suggesting that it may not be regulated by the SE region of LINC01013 (Fig. S1B). Subsequently, the interaction matrices from H3K27ac Hi-ChIP assay revealed more evident chromatin interactions for LINC01013 in hypoxia than in normoxia (Fig. 2A, left), and showed SE had direct interactions with the promoter region of LINC01013 to form a specific chromatin loop in hypoxia (Fig. 2A, right). Then, the subcellular localization of LINC01013 was detected by RNA fluorescence in situ hybridization (RNA-FISH) and confirmed by nuclear/cytosol fractionation experiment. We found that the expression of LINC01013 in hPASMCs was predominantly accumulated in the cytoplasm under hypoxia (Fig. 2B and C). Importantly, we also observed the distribution of LINC01013 in the mitochondria of hPASMCs, using TOM20 (Translocase of Outer Mitochondrial Membrane 20) as a mitochondrial marker (Fig. 2D). Collectively, these results highlight that LINC01013 is a novel hypoxia specific SE-driven lncRNA related to mitochondria.

### SE-associated LINC01013 is activated by CEBPB in hypoxia

**Fig. 3:**
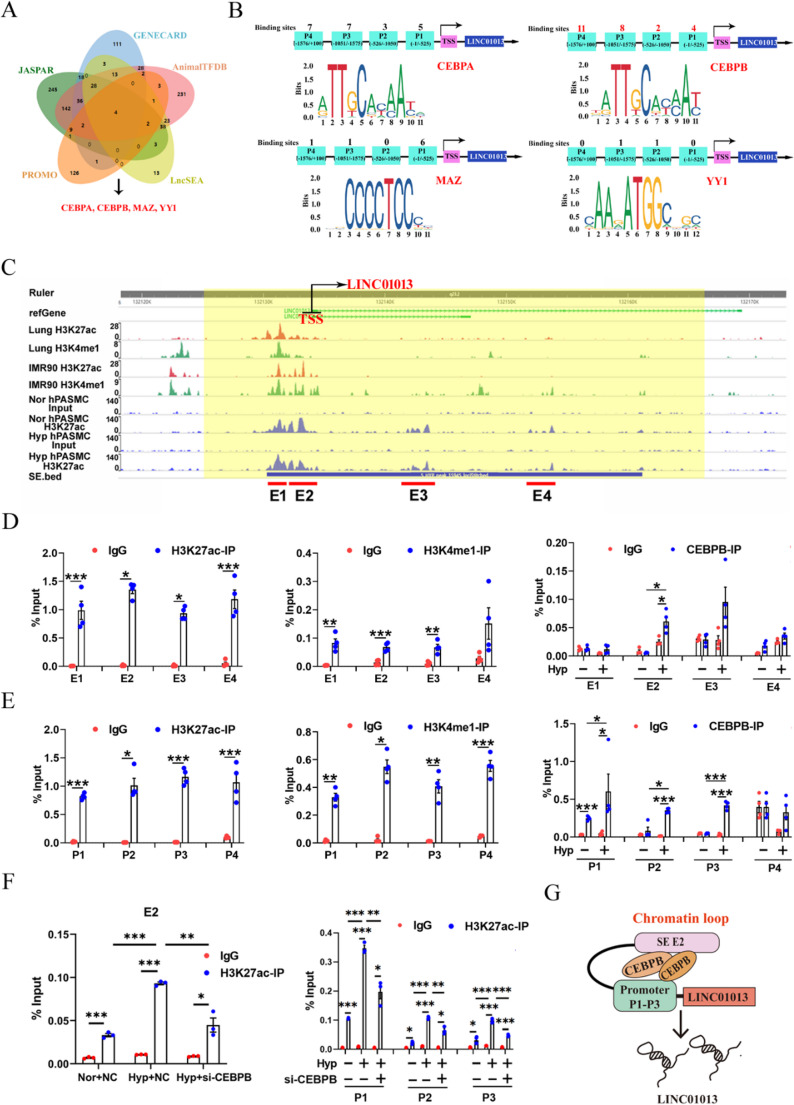
SE-associated LINC01013 was transcriptionally activated by CEBPB in hPASMCs.** A** Prediction of candidate transcription factors and binding sites. (Transcription factor related databases: JASPAR: https://jaspar.elixir.no/; PROMO: https://alggen.lsi.upc.es/cgi-bin/promo_v3/promo/promoinit.cgi?dirDB=TF_8.3; GENECARD: https://www.genecards.org/; AnimalTFDB: http://bioinfo.life.hust.edu.cn/HumanTFDB/#!/; Super enhancer related database: LncSEA: https://bio.liclab.net/LncSEA/). **B** The schematic diagram illustrates the LINC01013 promoter, divided into segments P1-P4, and the binding sites of transcription factors. **C** Four constituents (E1-E4) of SE region of LINC01013 derived from the WashU Epigenome Browser databases (http://epigenomegateway.wustl.edu/browser/). **D**,** E** hPASMCs were subjected to ChIP analysis using antibodies against H3K27ac, H3K4me1 and CEBPB. The association with the SE region (D, E1-E4) and promoter region (E, P1-P4) of LINC01013 was quantified by RT‒qPCR (*n* = 3). **F** hPASMCs were treated with CEBPB siRNA and subjected to ChIP analysis using antibodies against H3K27ac. The association with the E2 of SE (left) and P1-P3 promoter regions (right) of LINC01013 was quantified by RT-qPCR (*n* = 3). **G** Schematic diagram of transcribing LINC01013 in hPASMCs. All values are presented as the mean ± SEM. Statistical analysis was performed with one-way ANOVA or Student’s t-test. *p < 0.05, **p < 0.01, ***p < 0.001. Nor, normoxia; Hyp, hypoxia; NC, negative control; IP, immunoprecipitation; IgG, Immunoglobulin G; TSS, transcription initiation site

To gain an insight into how hypoxia activates LINC01013 by SE, we first identified candidate transcription factors that could bind to both of SE and promoter region of LINC01013, and the transcription factors including CEBPA, CEBPB, MAZ and YY1 were noted (Fig. 3A). Among them, CEBPB occupied more binding sites in LINC01013 promoter, which was likely the primary transcription regulator (Fig. 3B). Western blotting results showed that the expression level of CEBPB was upregulated in hypoxic hPASMCs (Fig S1C). As expected, CEBPB silencing inhibited hypoxia-mediated upregulation LINC01013 (Fig S1D and S1E). We further verified the interaction of CEBPB with H3K4me1 and H3K27ac by performing Co-IP assays (Fig S1F). Thereafter, the SE region of LINC01013 was divided into four constituents (E1–E4) according to monomethyl H3K4 (H3K4me1) and H3K27ac signaling peaks in the SE region of hPASMCs, human lung tissue and primary lung cells (IMR90) derived from the WashU Epigenome Browser databases (Fig. 3C). Using ChIP-qPCR, we validated that H3K27ac enriched in E1-E4 and H3K4me1 enriched in E1-E3, and hypoxia increased the binding of CEBPB and E2 specifically in hPASMCs, indicating E2 as a regulation region of the LINC01013 SE (Fig. 3D). Next, we further divided the promoter region of LINC01013 into four constituents (P1-P4) equally (Fig. 3B) and the ChIP-qPCR results showed a significant association of H3K27ac and H3K4me1 with the promoter regions of LINC01013, and hypoxia increased the recruitment of CEBPB to the P1-P3 regions of LINC01013 in hPASMCs (Fig. 3E). Meanwhile, CEBPB knockdown inhibited H3K27ac enrichment at the E2 and P1-P3 regions of LINC01013 (Fig. 3F). These data demonstrate that CEBPB co-occupy the promoter P1-P3 region and SE E2 region of LINC01013, thereby activating its transcription in hypoxic hPASMCs (Fig. 3G).

### LINC01013 promotes hypoxia-induced hPASMC proliferation and inflammation

**Fig. 4:**
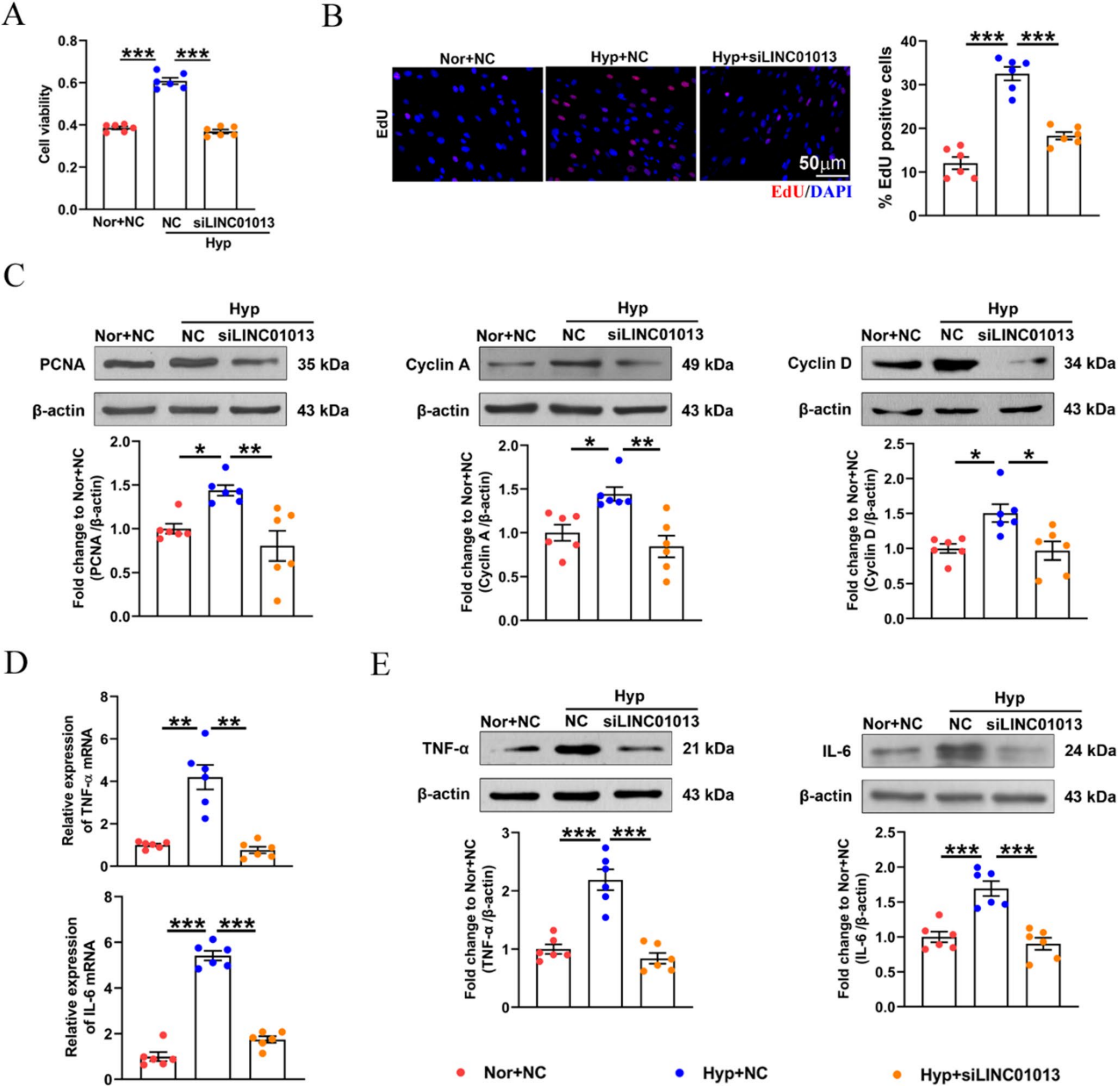
Silencing of LINC01013 inhibited hypoxia-induced hPASMC proliferation and inflammation.** A**,** B** hPASMC proliferation was determined by CCK8 and EdU incorporation assays (*n* = 6). EdU (red), DAPI (blue), scale bar, 50 μm. **C** Western blotting analysis of PCNA, cyclin A and cyclin D in hPASMCs (*n* = 6). **D** RT‒qPCR analysis showed the mRNA levels of TNF-α and IL-6 after LINC01013 knockdown, β-actin was used as a internal reference gene (*n* = 6). **E** Western blotting analysis of TNF-α and IL-6 in hPASMCs. (*n* = 6). All values are presented as the mean ± SEM. Statistical analysis was performed with one-way ANOVA. *p < 0.05, **p < 0.01, ***p < 0.001. Nor, normoxia; Hyp, hypoxia; NC, negative control; si, siRNA

To explore the function of LINC01013 in hPASMCs, we first validated the efficiency of LINC01013 knockdown via RT-qPCR (Fig S2). Next, CCK8 and EdU assays showed that transfection of LINC01013 siRNA inhibited hypoxia-induced proliferation of hPASMCs (Fig. 4A and B). Acordingly, the expression of PCNA, and cell cycle-related proteins (Cyclin A and Cyclin D) in hPASMCs were increased significantly under hypoxic conditions, which were reversed following LINC01013 siRNA (Fig. 4C). Additionally, we tried to investigate the role of LINC01013 in regulating hPASMC inflammation and found that compared to hypoxia, cells transduced with siLINC01013 showed decreased expression of TNF-α and IL-6, demonstrating that LINC01013 promotes the inflammatory phenotype of hPASMCs under hypoxic condition (Fig. 4D and E). To investigate the role of LINC01013 under normoxic conditions, we generated an LINC01013 overexpression construct and confirmed its efficiency by RT-qPCR (Fig S3A). We then assessed cell proliferation in hPASMCs and found that LINC01013 overexpression significantly enhanced cell viability, EdU incorporation, and PCNA expression (Fig S3B-S3D). Additionally, LINC01013 overexpression increased the expression of active inflammatory cytokines, including TNF-α and IL-6 in hPASMCs (Fig S3E). These results indicate that LINC01013 plays a key role in regulating hPASMC function.

### LINC01013 controls mitochondrial function

**Fig. 5:**
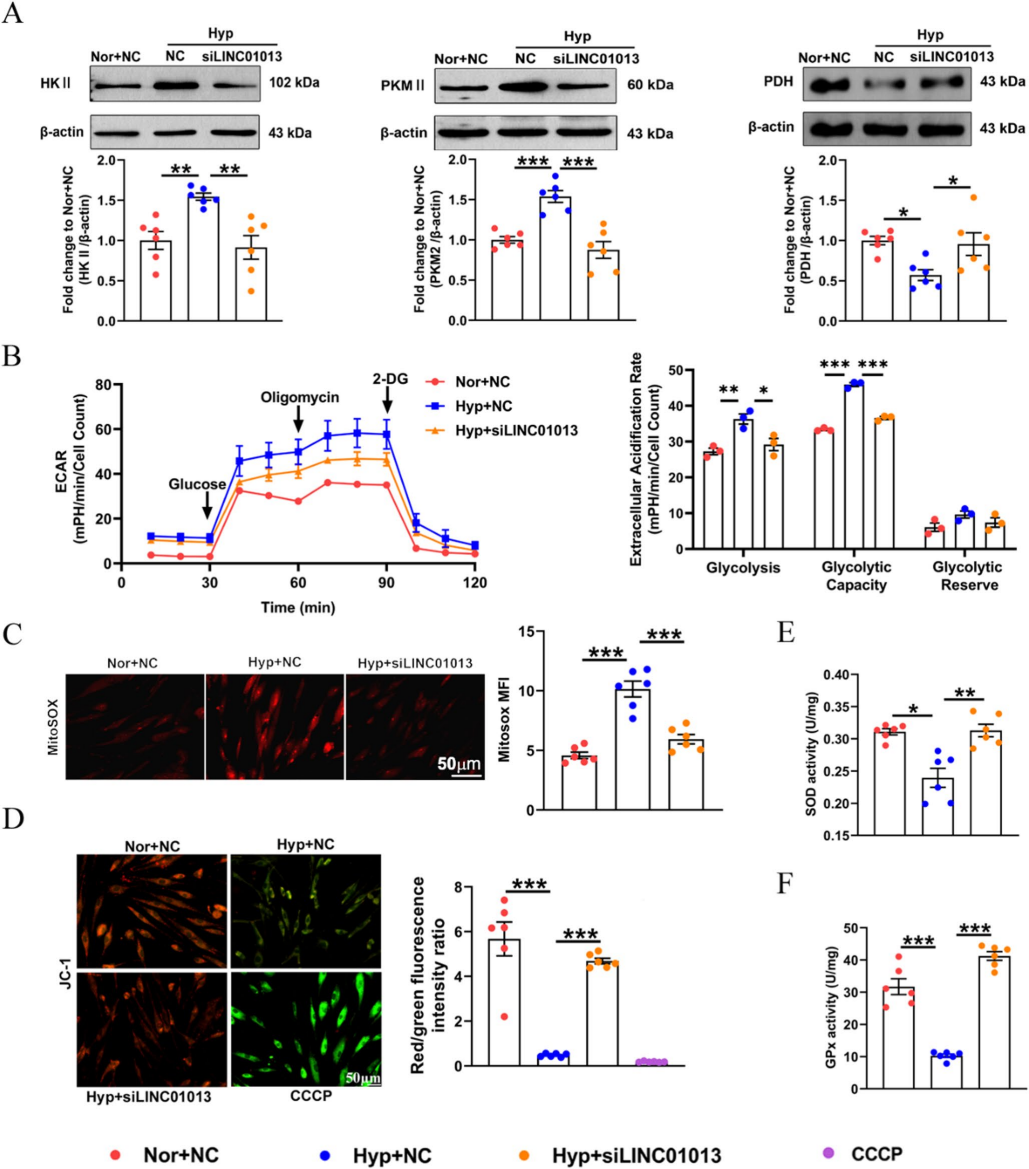
Silencing of LINC01013 inhibited hypoxia-induced hPASMC mitochondrial dysfunction.** A** HK II, PKM2, and PDH protein levels were regulated by siLINC01013 (*n* = 6). **B** Extracellular acidification rate (ECAR) of hPASMCs was measured via the Seahorse XFe24 platform after knockdown of LINC01013 (*n* = 3). **C** Effects of LINC01013 on redox imbalance after mitochondrially targeted superoxide indicator (MitoSOX, red) staining (*n* = 6). Scale bars, 50 μm. **D** Immunostaining of JC-1 aggregates (red) and JC-1 monomers (green) indicated the effect of LINC01013 inhibition on MMP in hypoxia-treated hPASMCs, using CCCP (Mitochondrial oxidative phosphorylation uncoupler, 10µM) as a positive control to induce MMP loss. (*n* = 6). Scale bars, 50 μm. **E**,** F** Quantitive analysis of antioxidative indices (SOD [superoxide dismutase] and GPx [glutathione peroxidase] activities) in hPASMCs (*n* = 6). All values are presented as the mean ± SEM. Statistical analysis was performed with one-way ANOVA. *p < 0.05, **p < 0.01, ***p < 0.001. Nor, normoxia; Hyp, hypoxia; NC, negative control; si, siRNA; MFI, Mean Fluorescence Intensity; CCCP, Carbonyl Cyanide 3-Chlorophenylhydrazone

Because the majority of LINC01013 was located in the cytoplasm and mitochondria of hPASMCs as showed in Fig. 2B-D, we next focused on its potential functions related to mitochondria including metabolic changes and oxidative stress. Hypoxia upregulated the expression of HK II (hexokinase II) and PKM2 (pyruvate kinase 2) and decreased the expression of PDH (pyruvate dehydrogenase), which were reversed by LINC01013 knockdown (Fig. 5A). Moreover, the glycolytic stress activation stimulated by hypoxia was significantly decreased in hPASMCs after LINC01013 knockdown as examined by ECAR (extracellular acidification rate) (Fig. 5B). To investigate the role of LINC01013 in mitochondrial injury, we assessed mitochondrial reactive oxygen species (ROS) and membrane potential (MMP). Knockdown of LINC01013 significantly attenuated hypoxia-induced mitochondrial superoxide production, as indicated by reduced MitoSOX fluorescence intensity (Fig. 5C). MMP was assessed using JC-1 staining. In mitochondria with normal MMP, JC-1 forms aggregates that emit red fluorescence. When MMP is decreased, JC-1 remains in monomeric form within the mitochondrial matrix, resulting in green fluorescence [24]. The red-to-green fluorescence intensity ratio was used to evaluate changes in MMP following LINC01013 knockdown, using CCCP (Carbonyl Cyanide 3-Chlorophenylhydrazone) as a positive control to induce MMP loss. The results showed that hypoxia reduced the MMP of hPASMCs, an effect that was reversed by siLINC01013 treatment (Fig. 5D). SOD and GPx activities results further confirmed that LINC01013 silencing reversed cell mitochondrial oxidative stress injury caused by hypoxia (Fig. 5E and F). Furthermore, Western blotting analysis showed that LINC01013 overexpression in normoxia effectively increased HKII expression (Fig S3F). Real-time monitoring of glycolysis using the Seahorse XFe24 extracellular flux analyzer revealed that, as expected, LINC01013 overexpression enhanced both glycolysis and glycolytic capacity (Fig S3G). Similarly, LINC01013 overexpression promoted mitochondrial ROS generation and MMP impairment (Fig S3H-S3I). Taken together, these findings indicate that LINC01013 regulates mitochondrial dysfunction in hPASMCs under both normoxic and hypoxic conditions.

### LINC01013 interacts with HSPA9

**Fig. 6:**
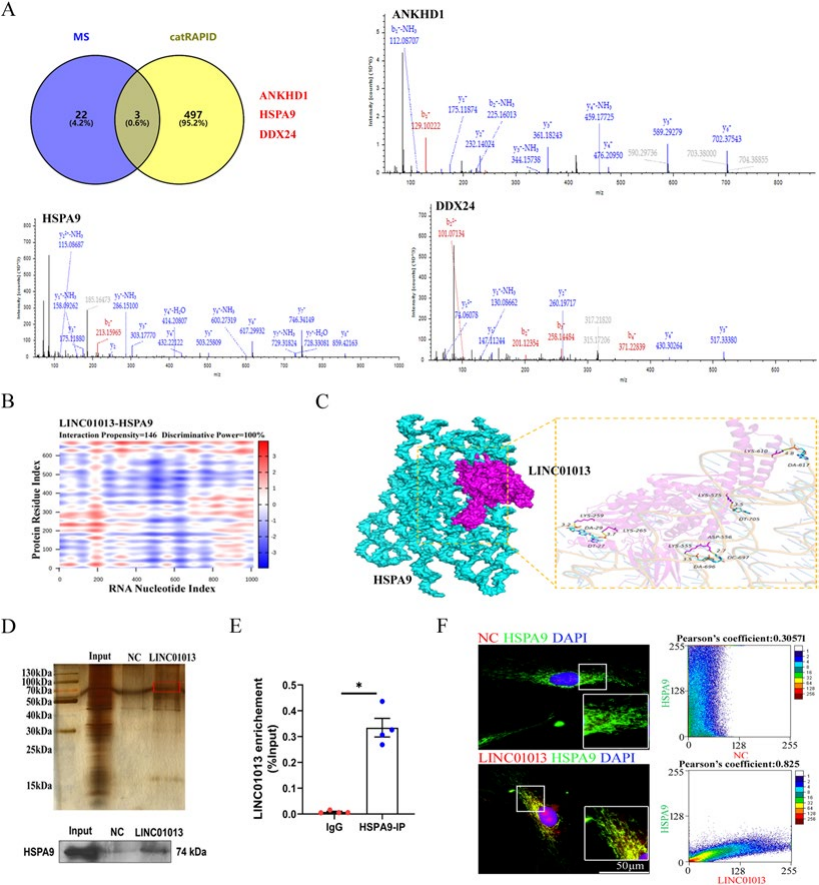
LINC01013 interacts with HSPA9.** A** ANKHD1, HSPA9 and DDX24 were identified as the candidate target proteins by catRAPID (http://s.tartaglialab.com/page/catrapid_group_old) and mass spectrometry (MS). **B** The heatmap results showed that LINC01013 can interact with the HSPA9 protein. **C** Schrodinger2019.01 software predicted and visualized the 3-dimensional structural docking of LINC01013 and HSPA9. **D** RNA pull-down followed by western blotting assays were used to detect the interaction between LINC01013 and the HSPA9 protein. **E** RNA immunoprecipitation (RIP) experiments demonstrated the association of HSPA9 with LINC01013 (*n* = 4). **F** RNA-FISH and immunofluorescence assay were used to observe the colocalization of LINC01013 (red) and HSPA9 protein (green). The Pearson correlation coefficient obtained through ImageJ reflects the correlation between the 2 fluorescence labels in colocalization, a non-targeting FISH probe was used as a negative control (NC). Scale bar, 50 μm. All values are presented as the mean ± SEM. Statistical analysis was performed with Student’s t-test. *p < 0.05. NC, negative control; IP, immunoprecipitation; IgG, Immunoglobulin G

To investigate the molecular mechanism underlying LINC01013 mediated mitochondrial homeostasis disruption in hypoxia, we designed a specific biotin-labeled LINC01013 probe to perform an RNA pulldown assay followed by protein mass spectrometry in hPASMCs. We screened target proteins including ankyrin repeat and KH domain containing 1 (ANKHD1), HSPA9 and DEAD-box helicase 24 (DDX24) interacted with LINC01013 based on protein mass spectrometry analysis and catRAPID prediction (Fig. 6A). Considering that LINC01013 played a major role in the regulation of mitochondrial function, our study first focused on the mitochondrial-localized protein, HSPA9. The interaction of LINC01013 with HSPA9 was further predicted by catRAPID, and the analysis revealed that LINC01013 may bound to HSPA9, and at HSPA9 476–564 nucleotide positions with high propensities (Fig. 6B, Fig S4A). Then, we visualized the 3-dimensional structural docking of 476–564 nucleotide positions, which support a model whereby LINC01013 and HSPA9 formed a stable RNA-protein complex (Fig. 6C). To clarify the possibility that LINC01013 interacts with HSPA9, Western blotting after RNA pulldown and RIP assays were performed, and the results confirmed that LINC01013 interact with HSPA9 (Fig. 6D and E). Consistent with these results, RNA-FISH assay and immunofluorescence staining showed that LINC01013 colocalized with HSPA9, with a Pearson correlation coefficient close to 1 (Fig. 6F). Moreover, immunofluorescence result showed that the expression of HSPA9 was upregulated, and localized in mitochondria in hypoxic hPASMCs (Fig S4B). However, as shown in Figure S4C, the expression of HSPA9 was not affected when LINC01013 was knocked down.

### LINC01013 cooperate with HSPA9 to impair mitochondrial homeostasis through VDAC1 oligomerization

**Fig. 7:**
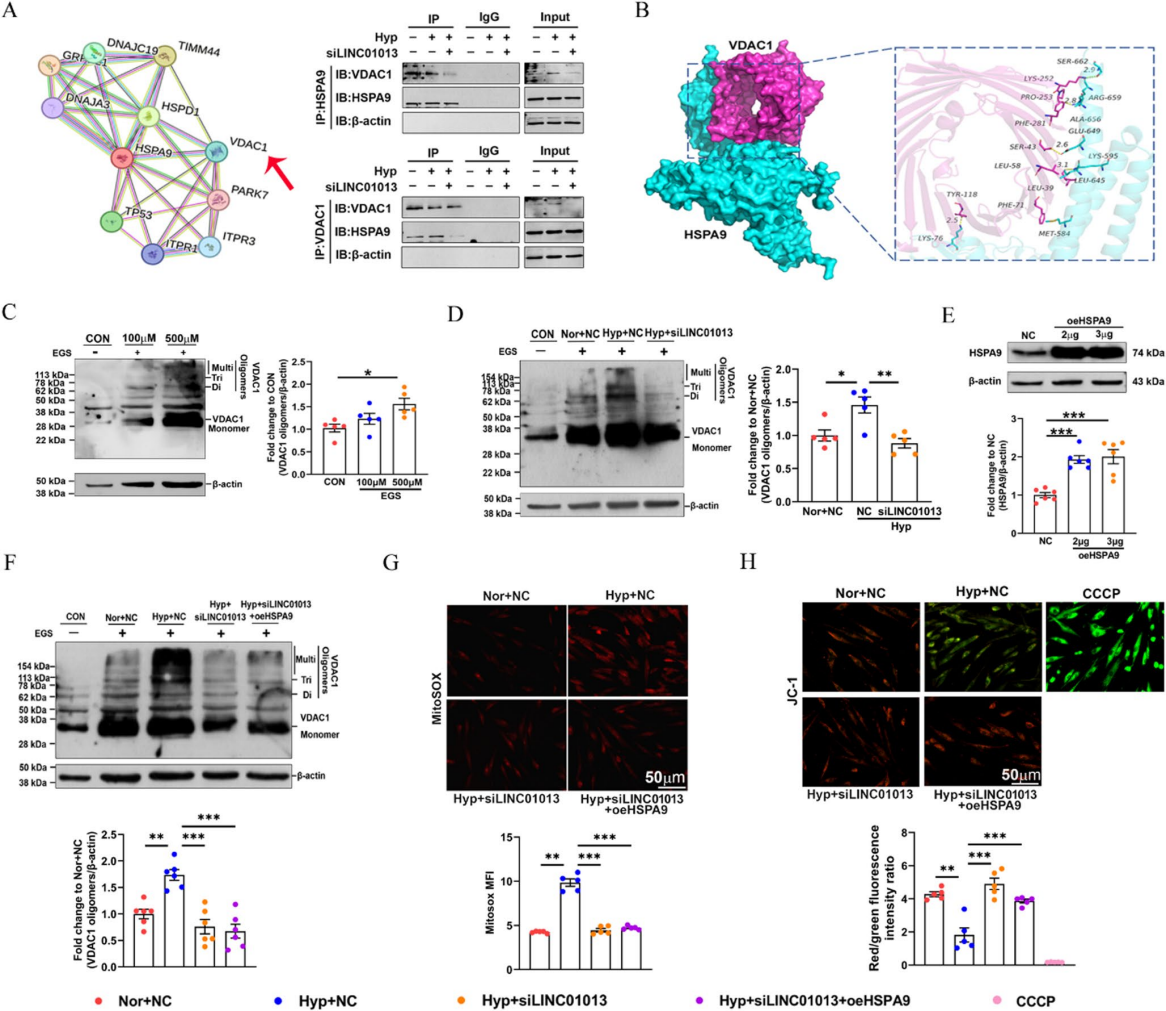
LINC01013 disrupts mitochondrial homeostasis by scaffolding the RNA-binding protein HSPA9-mediated VDAC1 oligomerization under hypoxia exposure. **A** Proteins possible interacting with HSPA9 were predicted from STRING database (https://cn.string-db.org/) (left). Coimmunoprecipitation (Co-IP) assay confirmed that LINC01013 knockdown affected the binding of HSPA9 and VDAC1 (right). **B** Schrodinger2019.01 software predicted and visualized the 3-dimensional structural docking of VDAC1 and HSPA9. **C** Effect of EGS on monomer and oligomers of VDAC1 in hPASMCs. The crosslinking reagent EGS was used to stabilize the oligomers during electrophoresis (*n* = 5) **D** Western blotting of VDAC1 monomer and oligomers in hPASMCs (*n* = 5). **E** Overexpression efficiency of HSPA9 were quantified by Western blotting (*n* = 6). **F** Effects of LINC01013 and HSPA9 on VDAC1 oligomerization. **G** Representative images showing mitochondrial reactive oxygen species (MitoSOX, red) staining in hPASMCs (*n* = 5). Scale bars, 50 μm. **H** Immunostaining of JC-1 aggregates (red) and monomers (green) showing changes in MMP across different groups. CCCP (10µM) was used as a positive control to induce MMP loss (*n* = 5). Scale bars, 50 μm. All values are presented as the mean ± SEM. Statistical analysis was performed with one-way ANOVA. *p < 0.05, **p < 0.01, ***p < 0.001. Nor, normoxia; Hyp, hypoxia; NC, negative control; si, siRNA; oe, overexpression; MFI, Mean Fluorescence Intensity; CCCP, Carbonyl Cyanide 3-Chlorophenylhydrazone

To investigate the role of LINC01013-HSPA9 complex in mitochondrial homeostasis of hPASMCs, we utilized the STRING database to search proteins interacting with HSPA9 and VDAC1 was identified, known for its pivotal roles to serve as a mitochondrial gatekeeper (Fig. 7A, left). We then examined the endogenous interaction between HSPA9 and VDAC1 through Co-IP assays and found that LINC01013 knockdown attenuated HSPA9-VDAC1 interactions, suggesting that LINC01013 acts as a protein scaffold to promote HSPA9 binding with VDAC1 under hypoxic conditions (Fig. 7A, right). The 3-dimensional structural docking result further demonstrated that HSPA9 and VDAC1 have multiple binding sites (Fig. 7B). Recent studies indicate that VDAC1 oligomerizes and forms large mitochondrial outer membrane permeabilization pores in the face of pathological stimuli and oxidative stress [25]. To this end, we examined the oligomerization state of VDAC1 by immunoblotting following treatment with the 100 µM and 500 µM cross-linking reagent Ethylene glycolbis (EGS) in hPASMCs, and the result showed that 500 µM EGS can stabilize the oligomers of VDAC1 (Fig. 7C). We then assessed the effects of LINC01013 on the oligomerization of VDAC1, revealing via Western blotting that LINC01013 knockdown inhibited VDAC1 oligomerization induced by hypoxia (Fig. 7D) To further explore the effect of the LINC01013-HSPA9 complex on the mitochondrial homeostasis, we attempted to gain insight into the functional recovery experiments with HSPA9 overexpression (Fig. 7E). However, Fig. 7F shows that transfection with LINC01013 siRNA reversed hypoxia induced oligomerization of VDAC1 in hPASMCs, while the effect was not eliminated after overexpression of HSPA9. MMP detection and mitochondrial ROS analysis further demonstrated that hypoxia-induced hPASMC mitochondrial injury could not be aggravated by HSPA9 overexpression when LINC01013 was knockdown, indicating that hypoxia-induced VDAC1 oligomerization by HSPA9 was in a LINC01013 depended manner (Fig. 7G and H). Altogether, our data demonstrate that LINC01013 disrupts mitochondrial homeostasis by scaffolding the RNA-binding protein HSPA9-mediated VDAC1 oligomerization under hypoxia exposure.

### LINC01013 promotes PH in mice

**Fig. 8:**
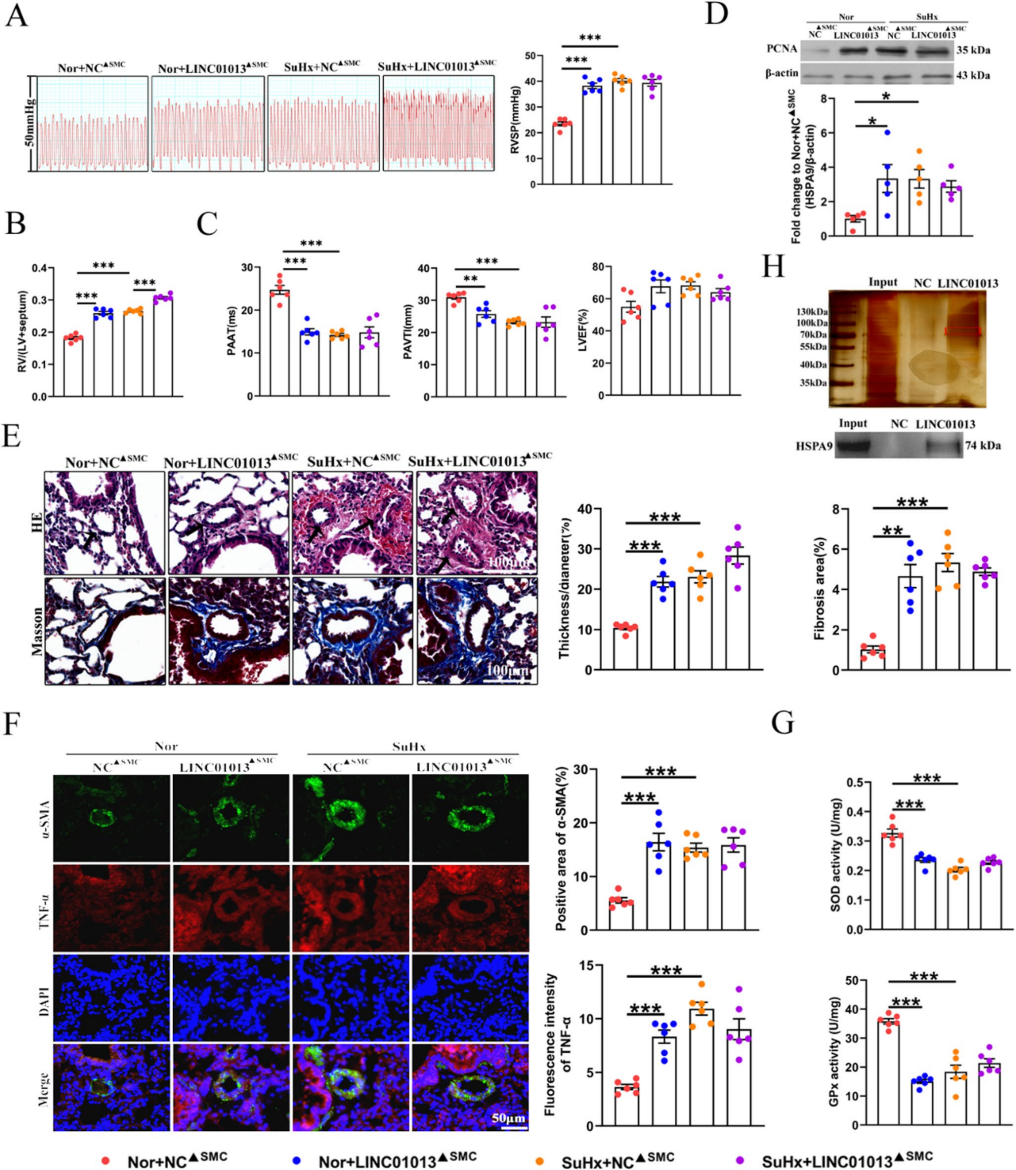
Human LINC01013 delivery aggravates PH progression in mice.** A**-**B** Right ventricular systolic pressure (RVSP) and RV/left ventricular (LV) + Septum weight ratio in the SuHx-induced PH mouse models (*n* = 6). **C** Pulmonary artery velocity time integral (PAVTI), pulmonary artery acceleration time (PAAT) and left ventricular ejection fraction (LVEF) of the SuHx-induced PH mice models infected with AAV5 carrying human LINC01013 (*n* = 6). **D** Expression of PCNA in lung tissues (*n* = 5). **E** Pulmonary arterial morphological analysis was performed by using hematoxylin and eosin (HE) and Masson staining. Scale bar, 100 μm. **F-G** Immunofluorescence of TNF-α, and activities of SOD and Gpx in lung tissues (*n* = 6). Scale bars, 50 μm. **H** RNA pull-down assays detected the interaction between LINC01013 and HSPA9 protein in mouse PASMCs. All values are presented as the mean ± SEM. Statistical analysis was performed with one-way ANOVA. *p < 0.05, **p < 0.01, ***p < 0.001. Nor, normoxia; SuHx, hypoxic + Su5416; NC, negative control; ▲SMC, smooth muscle cell targeting

To determine the effect of LINC01013 on pulmonary vascular remodeling in vivo, we established a SuHx-induced mouse model of PH and used AAV5-SM22α-LINC01013 to overexpress human LINC01013 specifically in the vascular smooth muscle layer (LINC01013^▲SMC^). To confirm the specificity of this intervention, we performed RNA-FISH and immunofluorescence staining, which revealed co-localization of LINC01013 with α-SMA in lung tissues, indicating successful targeted expression of LINC01013 in the vascular smooth muscle layer (Fig S5A). RT–qPCR analysis confirmed the successful delivery and increased expression of human LINC01013 in mouse lungs (Fig S5B). Additionally, Fig S5C shows that the protein sequences of HSPA9 are highly conserved between humans and mice. Ectopic expression of LINC01013 in normoxia substantially increased the right ventricular systolic pressure (RVSP) and right ventricle-to-left ventricle plus septum (RV/LV + S) ratio (Fig. 8A and B). According to echocardiographic measurements, LINC01013 decreased pulmonary artery velocity time integral (PAVTI) and pulmonary artery acceleration time (PAAT) in normoxic mice, similar to that of SuHx, while SuHx with LINC01013 overexpression did not further exacerbate this effect, and the left ventricular ejection fraction (LVEF) was not affected by LINC01013 (Fig. 8C). Similarly, we examined the effect of LINC01013 overexpression on cell proliferation in lung tissues, and found that the expression of PCNA was significantly increased when LINC01013 was overexpressed in normoxia, comparable to those of SuHx (Fig. 8D). We then performed pulmonary vascular morphological analysis by using hematoxylin–eosin (H&E) and Masson staining to show LINC01013 enhanced distal pulmonary vascular remodeling and collagenation (Fig. 8E). Consistently, immunofluorescence staining of TNF-α and the activities of SOD and GPx assays revealed that overexpression of LINC01013 promoted the inflammation and mitochondrial oxidative stress injury in lung tissues (Fig. 8F and G). Finally, human LINC01013 could bind to HSPA9 as determined in mouse PASMCs, indicating the effect of LINC01013 may also be achieved by interacting with HSPA9 in mouse PH models (Fig. 8H). Taken together, these results show that human LINC01013 delivery aggravates PASMC oxidative stress, inflammation and PH progression in mice.

## Discussion

**Fig. 9:**
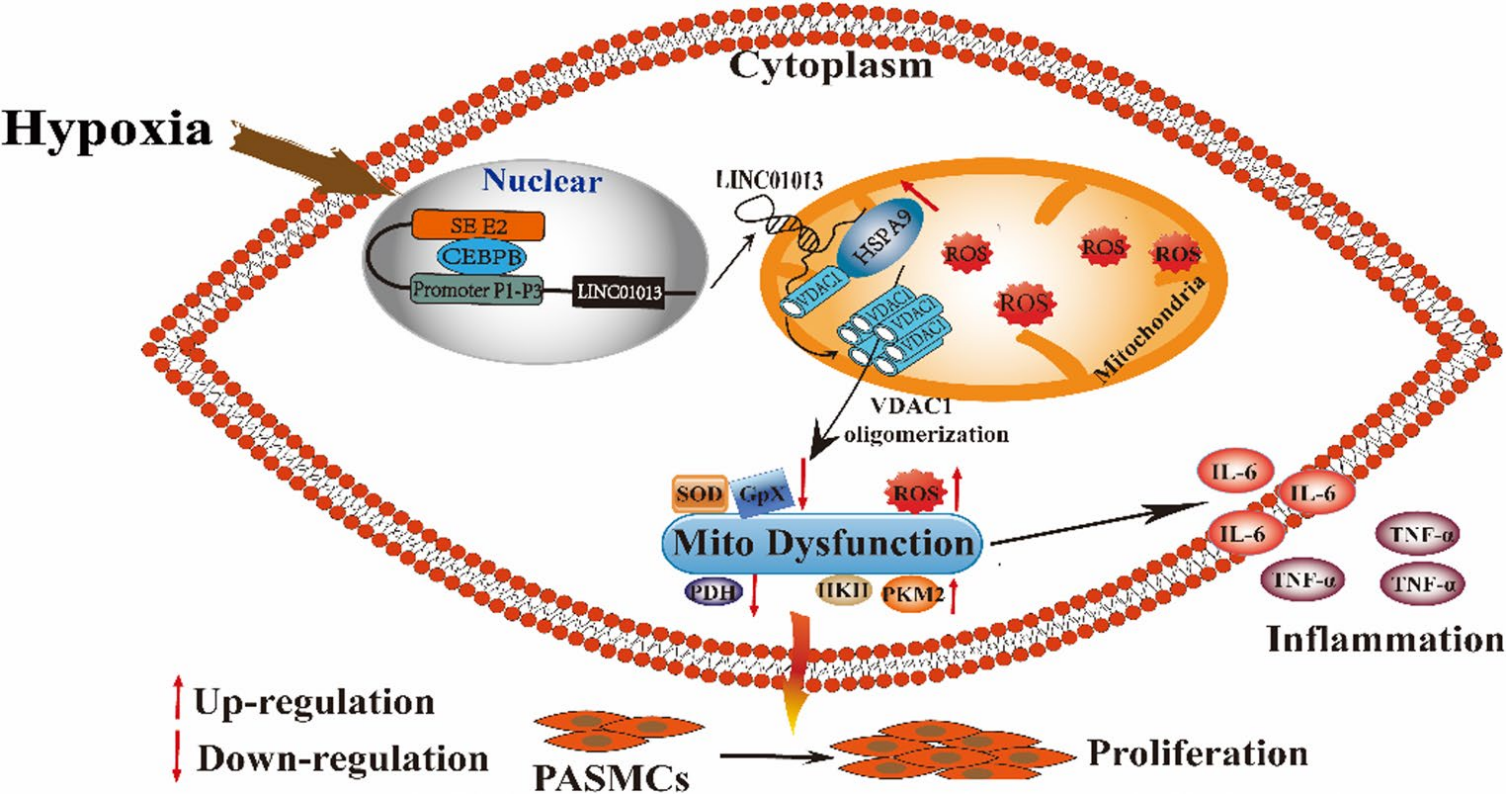
A schematic diagram illustrating the hypothetical role of LINC01013 in hypoxia-induced pulmonary arterial smooth muscle cell (PASMC) fate. In the nucleus, Ttranscription factor CEBPB directly bound SE and promoter region of LINC01013, and activated the transcription of LINC01013 in hypoxic PASMCs. In the mitochondria, LINC01013 as a protein-binding scaffold connecting HSPA9 and VDAC1, contributing to the oligomerization of VDAC1 and mitochondrial dysfunction by including oxidative stress and metabolic disorders, and ultimately leading to the proliferation and inflammation of PASMCs. SE, super-enhancer; ROS, reactive oxygen species; PASMCs, pulmonary arterial smooth muscle cells; HSPA9, heat shock protein family A (Hsp70) member 9; VDAC1, voltage dependent anion channel 1; CEBPB, CCAAT enhancer binding protein beta; SOD, superoxide dismutase; GPx, glutathione peroxidase; HK II, hexokinase 2; PKM2, pyruvate kinase M 2; PDH, pyruvate dehydrogenase

In the present study, we identified that hypoxia regulates LINC01013 transcription by recruiting CEBPB and SE to the LINC01013-promoter region, subsequently regulating mitochondria-dependent hPASMC proliferation and inflammation. We confirmed that LINC01013 as a protein-binding scaffold connecting HSPA9 and VDAC1, contributing to the oligomerization of VDAC1 and mitochondrial dysfunction including oxidative stress and metabolic disorders, and ultimately hypoxic PH progression (Fig. 9).

As hyperactive regulatory elements, SEs are clusters of activated enhancers capable of driving high levels of gene transcription [26]. Herein, we screened and identified that the SE-associated LINC01013 was highly expressed in hypoxic hPASMCs by ChIP-seq and ChIP-qPCR assay with antibody against H3K27ac. Moreover, we revealed that promoter (P1-P3) and SE2 of LINC01013 were occupied by H3K27ac, H3K4me1 and CEBPB to form a chromatin loop and activate its transcription, and Hi-ChIP assay further validated the specific chromatin interaction loop in hypoxia. CEBPB is an isoform in the family of multifunctional basic leucine zipper transcription factors, studies have demonstrated that CEBPB could affect the expression of SE–driving genes including phosphoenolpyruvate carboxykinase 1 (PCK1) and ephrin A1 (EFNA1) [27]. In our study, we screened and predicted transcription factors between SEs and promoter of LINC01013 and found that CEBPB as a major regulator that promotes transcription initiation of LINC01013 in hPASMCs. However, we cannot exclude the role of CEBPA, MAZ and YY1 as well as other transcription factors are involved in the regulation of LINC01013 transcription. The transcriptional profile of LINC01013 remains to be fully elucidated and would be valuable to characterize in future studies.

Several reports have highlighted the important role of LINC01013 in the regulation of pathophysiology. For example, Arnaud et al. recently reported that LINC01013 promotes the fibrogenic program in calcific aortic valve disease via its association with SE [28]. Yang et al. demonstrated that knockdown of LINC01013 enhances the chondrogenic differentiation of stem cells from apical papilla, and promotes cartilage tissue regeneration [29]. Chung et al. showed that LINC01013 knockdown significantly suppresses anaplastic large-cell lymphoma cell invasion by regulating epithelial-to-mesenchymal transition [30]. Despite the aforementioned functions, the role of LINC01013 in PH remains unexplored. Here, our innovative findings showed that knockdown of LINC01013 significantly reversed hypoxia-induced PASMC proliferation and inflammation. Interestingly, LINC01013 was found to distribute in mitochondria after transcription, which leads us to speculate on the importance of LINC01013 in regulating mitochondrial function. We then measured metabolic changes and oxidative stress in hPASMCs, and the data demonstrated that knockdown of LINC01013 inhibits the glycolysis and ROS accumulation, and increases the SOD and GPx activities in hypoxia. Consistent with the present study, Barton et al. identified a small open reading frame (ORF) within LINC01013 encoding a micropeptide that localizes to the mitochondria. Their work further demonstrates that both LINC01013 and this encoded micropeptide are critical for fibroblast activation, suggesting their potential as novel therapeutic targets for myocardial fibrosis [31]. In contrast, Boon et al. reported that the interaction between LINC01013 and the Y-Box protein 1 (YBX1) is essential for activating DNA repair pathways. They also found that the loss of LINC01013 impairs endothelial function, leading to reduced angiogenesis and migration [32]. Given these cell-type-specific functions, further studies are warranted to explore the role of LINC01013 in other cell types during pulmonary vascular remodeling.

Our in vivo experiments demonstrated that LINC01013 plays a critical role in the progression of PH. Delivery of AAV5 carrying human LINC01013 into mice promoted PASMC proliferation, inflammation, and vascular remodeling through the regulation of mitochondrial function. These findings highlight the biological importance of SE-driven LINC01013 as a key regulator of hypoxia-induced mitochondrial dysfunction and its role in governing pulmonary vascular remodeling. Although we have identified a mouse homolog of HSPA9 (a binding partner of LINC01013) that is highly conserved between humans and mice, our study is limited by the absence of a murine ortholog for LINC01013 itself. This evolutionary divergence precludes the use of conventional mouse models for smooth muscle cell-specific knockout of LINC01013 to conduct functional validation studies. The species-specific differences between mice and humans in terms of immune response, mitochondrial function, and cellular pathways may affect the translatability of our findings. Moreover, the absence of clinical samples from patients with PH further limits the clinical translation of this research. Future studies using alternative models—such as organoids or humanized mouse models—will help to better evaluate the therapeutic potential of LINC01013.

HSPA9, a member of the heat shock protein 70 (HSP70) family, predominantly localizes in mitochondria and is aberrantly overexpressed in several mitochondria-related diseases. In glioblastoma, HSPA9 interacts with OMA1 zinc metallopeptidase (OMA1) and induces mitophagy [33]. HSPA9 was also reported to promote proliferation of medullary thyroid carcinoma cells through regulating mitochondrial bioenergetics [34]. In this study, we identified HSPA9 as a novel protein interactor of LINC01013 in PASMCs, and found that the expression of HSPA9 was triggered by hypoxia during mitochondrial dysfunction. Unexpectedly, the protein expression of HSPA9 was not affected by LINC01013, suggesting there are additional ways of LINC01013 to mediate mitochondrial dysfunction. Studies have shown that VDAC1 is physically interacts with HSPA9 to play a role in mitochondrial calcium overload and diabetic atrial remodeling. Indeed, we showed that HSPA9 overexpression could increase VDAC1 oligomerization in hPASMCs, and siLINC01013 reversed the effect induced by hypoxia.

VDAC1 is located in the outer membrane of mitochondria and controls the exchange of metabolites, nucleotides and ions between mitochondria and cytosol. Aberrant expression and modification of VDAC1 can participate in the development of diseases by regulating various cytopathological processes, such as mitochondrial outer membrane permeabilization, mitochondrial DNA release and mtROS production [25, 35]. Wu et al. reported that ubiquitination of VDAC1 at a specific site confer protection against liver fibrosis by inhibiting mtDNA release [36]. Zhou et al. revealed that transmembrane BAX inhibitor motif containing 6 (TMBIM6)-VDAC1 interaction prevent VDAC1 oligomerization to improve myocardial function in septic cardiomyopathy [37]. Specifically, in our study, LINC01013 knockdown significantly alleviated mitochondrial dysfunction, cell proliferation and vascular remodeling under hypoxic conditions, which is most likely achieved through HSPA9 and VDAC1. Mechanistically, we propose a model for the HSPA9-dependent VDAC1 oligomerization where LINC01013-scaffold plays a central role in linking HSPA9 and VDAC1 complex. The finding provides a new insight for LINC01013 involved in mitochondrial dysfunction in hPASMCs by interacting with the target protein rather than affecting its expression. However, our study did not clarify the specific binding region of LINC01013 with HSPA9 and VDAC1, which is another limitation of this study. Our findings report a SE-associated lncRNA, LINC01013, that is activated by CEBPB to regulate mitochondrial dysfunction by interacting with the HSPA9/VDAC1 axis. The finding implies that LINC01013 may serve as a potential novel target of nucleic acids to treat PASMC proliferation and inflammation in PH.

## Supplementary Information

Below is the link to the electronic supplementary material.

Supplementary Material 1

Supplementary Material 2

## Data Availability

All data generated or analyzed during this study are included in this published article and its supplementary information files.

## References

[1] Johnson S, Sommer N, Cox-Flaherty K, Weissmann N, Ventetuolo CE, Maron BA (2023) Pulmonary hypertension: A contemporary review. Am J Respir Crit Care Med 208(5):528–548 37450768 10.1164/rccm.202302-0327SO PMC10492255

[2] Humbert M, Guignabert C, Bonnet S, Dorfmuller P, Klinger JR, Nicolls MR, Olschewski AJ, Pullamsetti SS, Schermuly RT, Stenmark KR, Rabinovitch M (2019) Pathology and pathobiology of pulmonary hypertension: state of the Art and research perspectives. Eur Respir J 53(1):1801887 30545970 10.1183/13993003.01887-2018 PMC6351340

[3] Song S, Carr SG, McDermott KM, Rodriguez M, Babicheva A, Balistrieri A, Ayon RJ, Wang J, Makino A, Yuan JX (2018) STIM2 (Stromal interaction molecule 2)-Mediated increase in resting cytosolic free Ca(2+) concentration stimulates PASMC proliferation in pulmonary arterial hypertension. Hypertension 71(3):518–529 29358461 10.1161/HYPERTENSIONAHA.117.10503 PMC5955710

[4] Zhang M, Xin W, Yu Y, Yang X, Ma C, Zhang H, Liu Y, Zhao X, Guan X, Wang X, Zhu D (2020) Programmed death-ligand 1 triggers PASMCs pyroptosis and pulmonary vascular fibrosis in pulmonary hypertension. J Mol Cell Cardiol 138:23–33 31733200 10.1016/j.yjmcc.2019.10.008

[5] Feng W, Wang J, Yan X, Zhang Q, Chai L, Wang Q, Shi W, Chen Y, Liu J, Qu Z, Li S, Xie X, Li M (2021) ERK/Drp1-dependent mitochondrial fission contributes to HMGB1-induced autophagy in pulmonary arterial hypertension. Cell Prolif 54(6):e13048 33948998 10.1111/cpr.13048 PMC8168414

[6] He S, Bai J, Zhang L, Yuan H, Ma C, Wang X, Guan X, Mei J, Zhu X, Xin W, Zhu D (2024) Superenhancer-driven circrna Myst4 involves in pulmonary artery smooth muscle cell ferroptosis in pulmonary hypertension. iScience 27(10):110900 39351203 10.1016/j.isci.2024.110900 PMC11440257

[7] Ruffenach G, Chabot S, Tanguay VF, Courboulin A, Boucherat O, Potus F, Meloche J, Pflieger A, Breuils-Bonnet S, Nadeau V, Paradis R, Tremblay E, Girerd B, Hautefort A, Montani D, Fadel E, Dorfmuller P, Humbert M, Perros F, Paulin R, Provencher S, Bonnet S (2016) Role for Runt-related transcription factor 2 in proliferative and calcified vascular lesions in pulmonary arterial hypertension. Am J Respir Crit Care Med 194(10):1273–1285 27149112 10.1164/rccm.201512-2380OC

[8] Lu X, Zhang J, Liu H, Ma W, Yu L, Tan X, Wang S, Ren F, Li X, Li X (2021) Cannabidiol attenuates pulmonary arterial hypertension by improving vascular smooth muscle cells mitochondrial function. Theranostics 11(11):5267–5278 33859746 10.7150/thno.55571 PMC8039951

[9] Liu M, He H, Fan F, Qiu L, Zheng F, Guan Y, Yang G, Chen L (2023) Maresin-1 protects against pulmonary arterial hypertension by improving mitochondrial homeostasis through ALXR/HSP90alpha axis. J Mol Cell Cardiol 181:15–30 37244057 10.1016/j.yjmcc.2023.05.005

[10] Yan X, Huang J, Zeng Y, Zhong X, Fu Y, Xiao H, Wang X, Lian H, Luo H, Li D, Guo R (2024) CGRP attenuates pulmonary vascular remodeling by inhibiting the cGAS-STING-NFkappaB pathway in pulmonary arterial hypertension. Biochem Pharmacol 222:116093 38408681 10.1016/j.bcp.2024.116093

[11] Ma C, Zhang C, Ma M, Zhang L, Zhang L, Zhang F, Chen Y, Cao F, Li M, Wang G, Shen T, Yao H, Liu Y, Pan Z, Song S, Zhu D (2017) MiR-125a regulates mitochondrial homeostasis through targeting Mitofusin 1 to control hypoxic pulmonary vascular remodeling. J Mol Med (Berl) 95(9):977–993 28593577 10.1007/s00109-017-1541-5

[12] Ma C, Wang X, He S, Zhang L, Bai J, Qu L, Qi J, Zheng X, Zhu X, Mei J, Guan X, Yuan H, Zhu D (2022) Ubiquitinated AIF is a major mediator of hypoxia-induced mitochondrial dysfunction and pulmonary artery smooth muscle cell proliferation. Cell Biosci 12(1):9 35090552 10.1186/s13578-022-00744-3 PMC8796423

[13] Han Y, Liu Y, Yang C, Gao C, Guo X, Cheng J (2020) LncRNA CASC2 inhibits hypoxia-induced pulmonary artery smooth muscle cell proliferation and migration by regulating the miR-222/ING5 axis. Cell Mol Biol Lett 25:21 32206065 10.1186/s11658-020-00215-y PMC7079380

[14] Liu Y, Zhang H, Li Y, Yan L, Du W, Wang S, Zheng X, Zhang M, Zhang J, Qi J, Sun H, Zhang L, Li G, Zhu D (2020) Long noncoding RNA Rps4l mediates the proliferation of hypoxic pulmonary artery smooth muscle cells. Hypertension 76(4):1124–1133 32772647 10.1161/HYPERTENSIONAHA.120.14644

[15] Wang X, Li Q, He S, Bai J, Ma C, Zhang L, Guan X, Yuan H, Li Y, Zhu X, Mei J, Gao F, Zhu D (2022) LncRNA FENDRR with m6A RNA methylation regulates hypoxia-induced pulmonary artery endothelial cell pyroptosis by mediating DRP1 DNA methylation. Mol Med 28(1):126 36284300 10.1186/s10020-022-00551-z PMC9594874

[16] Blayney JW, Francis H, Rampasekova A, Camellato B, Mitchell L, Stolper R, Cornell L, Babbs C, Boeke JD, Higgs DR, Kassouf M (2023) Super-enhancers include classical enhancers and facilitators to fully activate gene expression. Cell 186(26):5826-39 e18 10.1016/j.cell.2023.11.030 PMC10858684 38101409

[17] Li J, Zhu J, Gray O, Sobreira DR, Wu D, Huang RT, Miao B, Sakabe NJ, Krause MD, Kaikkonen MU, Romanoski CE, Nobrega MA, Fang Y (2024) Mechanosensitive super-enhancers regulate genes linked to atherosclerosis in endothelial cells. J Cell Biol 223(3):e202211125 38231044 10.1083/jcb.202211125 PMC10794123

[18] Thandapani P (2019) Super-enhancers in cancer. Pharmacol Ther 199:129–138 30885876 10.1016/j.pharmthera.2019.02.014

[19] Lakhia R, Mishra A, Biggers L, Malladi V, Cobo-Stark P, Hajarnis S, Patel V (2023) Enhancer and super-enhancer landscape in polycystic kidney disease. Kidney Int 103(1):87–99 36283570 10.1016/j.kint.2022.08.039 PMC9841439

[20] Su T, Zhang N, Wang T, Zeng J, Li W, Han L, Yang M (2023) Super Enhancer-Regulated LncRNA LINC01089 induces alternative splicing of DIAPH3 to drive hepatocellular carcinoma metastasis. Cancer Res 83(24):4080–4094 37756562 10.1158/0008-5472.CAN-23-0544

[21] Zhang Q, Song C, Zhang M, Liu Y, Wang L, Xie Y, Qi H, Ba L, Shi P, Cao Y, Sun H (2023) Super-enhancer-driven LncRNA Snhg7 aggravates cardiac hypertrophy via Tbx5/GLS2/ferroptosis axis. Eur J Pharmacol 953:175822 37277029 10.1016/j.ejphar.2023.175822

[22] Ma C, Wang X, Zhang L, Zhu X, Bai J, He S, Mei J, Jiang J, Guan X, Zheng X, Qu L, Zhu D (2023) Super Enhancer-Associated circular RNA-CircKrt4 regulates hypoxic pulmonary artery endothelial cell dysfunction in mice. Arterioscler Thromb Vasc Biol 43(7):1179–1198 37139839 10.1161/ATVBAHA.122.318842

[23] Ma C, Gu R, Wang X, He S, Bai J, Zhang L, Zhang J, Li Q, Qu L, Xin W, Jiang Y, Li F, Zhao X, Zhu D (2020) CircRNA CDR1as promotes pulmonary artery smooth muscle cell calcification by upregulating CAMK2D and CNN3 via sponging miR-7-5p. Mol Ther Nucleic Acids 22:530–541 33230455 10.1016/j.omtn.2020.09.018 PMC7566008

[24] Yu M, Song M, Zhang M, Chen S, Ni B, Li X, Lei W, Shen Z, Fan Y, Zhang J, Hu S (2025) Mitochondrial mutation leads to cardiomyocyte hypertrophy by disruption of Mitochondria-Associated ER membrane. Cell Prolif 58(7):e70002 39981966 10.1111/cpr.70002 PMC12240637

[25] Kim J, Gupta R, Blanco LP, Yang S, Shteinfer-Kuzmine A, Wang K, Zhu J, Yoon HE, Wang X, Kerkhofs M, Kang H, Brown AL, Park SJ, Xu X, van Zandee E, Kim MK, Cohen JI, Kaplan MJ, Shoshan-Barmatz V, Chung JH (2019) VDAC oligomers form mitochondrial pores to release MtDNA fragments and promote lupus-like disease. Science 366(6472):1531–1536 31857488 10.1126/science.aav4011 PMC8325171

[26] Whyte WA, Orlando DA, Hnisz D, Abraham BJ, Lin CY, Kagey MH, Rahl PB, Lee TI, Young RA (2013) Master transcription factors and mediator Establish super-enhancers at key cell identity genes. Cell 153(2):307–319 23582322 10.1016/j.cell.2013.03.035 PMC3653129

[27] Li H, Li J, Xiao T, Hu Y, Yang Y, Gu X, Jin G, Cao H, Zhou H, Yang C (2022) Nintedanib alleviates experimental colitis by inhibiting CEBPB/PCK1 and CEBPB/EFNA1 pathways. Front Pharmacol 13:904420 35910380 10.3389/fphar.2022.904420 PMC9331303

[28] Chignon A, Argaud D, Boulanger MC, Mkannez G, Bon-Baret V, Li Z, Theriault S, Bosse Y, Mathieu P (2022) Genome-wide chromatin contacts of super-enhancer-associated LncRNA identify LINC01013 as a regulator of fibrosis in the aortic valve. PLoS Genet 18(1):e1010010 35041643 10.1371/journal.pgen.1010010 PMC8797204

[29] Yang H, Cao Y, Zhang J, Liang Y, Su X, Zhang C, Liu H, Han X, Ge L, Fan Z (2020) DLX5 and HOXC8 enhance the chondrogenic differentiation potential of stem cells from apical papilla via LINC01013. Stem Cell Res Ther 11(1):271 32631410 10.1186/s13287-020-01791-8 PMC7336658

[30] Chung IH, Lu PH, Lin YH, Tsai MM, Lin YW, Yeh CT, Lin KH (2017) The long non-coding RNA LINC01013 enhances invasion of human anaplastic large-cell lymphoma. Sci Rep 7(1):295 28331184 10.1038/s41598-017-00382-7 PMC5428265

[31] Quaife NM, Chothani S, Schulz JF, Lindberg EL, Vanezis K, Adami E, O’Fee K, Greiner J, Litvinukova M, van Heesch S, Whiffin N, Hubner N, Schafer S, Rackham O, Cook SA, Barton PJR (2023) LINC01013 is a determinant of fibroblast activation and encodes a novel fibroblast-Activating micropeptide. J Cardiovasc Transl Res 16(1):77–85 35759180 10.1007/s12265-022-10288-z PMC9944705

[32] Pham TP, Bink DI, Stanicek L, van Bergen A, van Leeuwen E, Tran Y, Matic L, Hedin U, Wittig I, Dimmeler S, Boon RA (2020) Long Non-coding RNA Aerrie controls DNA damage repair via YBX1 to maintain endothelial cell function. Front Cell Dev Biol 8:619079 33505972 10.3389/fcell.2020.619079 PMC7829583

[33] Zhu W, Rao J, Zhang LH, Xue KM, Li L, Li JJ, Chen QZ, Fu R (2024) OMA1 competitively binds to HSPA9 to promote mitophagy and activate the cGAS-STING pathway to mediate GBM immune escape. J Immunother Cancer 12(4):e008718 38604814 10.1136/jitc-2023-008718 PMC11015223

[34] Starenki D, Hong SK, Lloyd RV, Park JI (2015) Mortalin (GRP75/HSPA9) upregulation promotes survival and proliferation of medullary thyroid carcinoma cells. Oncogene 34(35):4624–4634 25435367 10.1038/onc.2014.392 PMC4451452

[35] Belosludtsev KN, Serov DA, Ilzorkina AI, Starinets VS, Dubinin MV, Talanov EY, Karagyaur MN, Primak AL, Belosludtseva NV (2023) Pharmacological and genetic suppression of VDAC1 alleviates the development of mitochondrial dysfunction in endothelial and fibroblast cell cultures upon hyperglycemic conditions. Antioxidants (Basel) 12(7) 10.3390/antiox12071459 PMC10376467 37507997

[36] Wu NN, Wang L, Wang L, Xu X, Lopaschuk GD, Zhang Y, Ren J (2023) Site-specific ubiquitination of VDAC1 restricts its oligomerization and mitochondrial DNA release in liver fibrosis. Exp Mol Med 55(1):269–280 36658227 10.1038/s12276-022-00923-9 PMC9898252

[37] Zhou H, Dai Z, Li J, Wang J, Zhu H, Chang X, Wang Y (2023) TMBIM6 prevents VDAC1 multimerization and improves mitochondrial quality control to reduce sepsis-related myocardial injury. Metabolism 140:155383 36603706 10.1016/j.metabol.2022.155383

