# Supplementary material for: Super enhancer-driven LINC01013 mediates hypoxia-induced mitochondrial dysfunction by HSPA9 to determine pulmonary arterial smooth muscle cell fate
Source: Cell Mol Life Sci. 2026 Jan 6;83(1):57. doi: 10.1007/s00018-025-06071-3 (PMC12819934; doi:10.1007/s00018-025-06071-3)
Supplement: Supplementary file 1 — Supplementary Material 1 [file 18_2025_6071_MOESM1_ESM.docx]

**Fig S1**


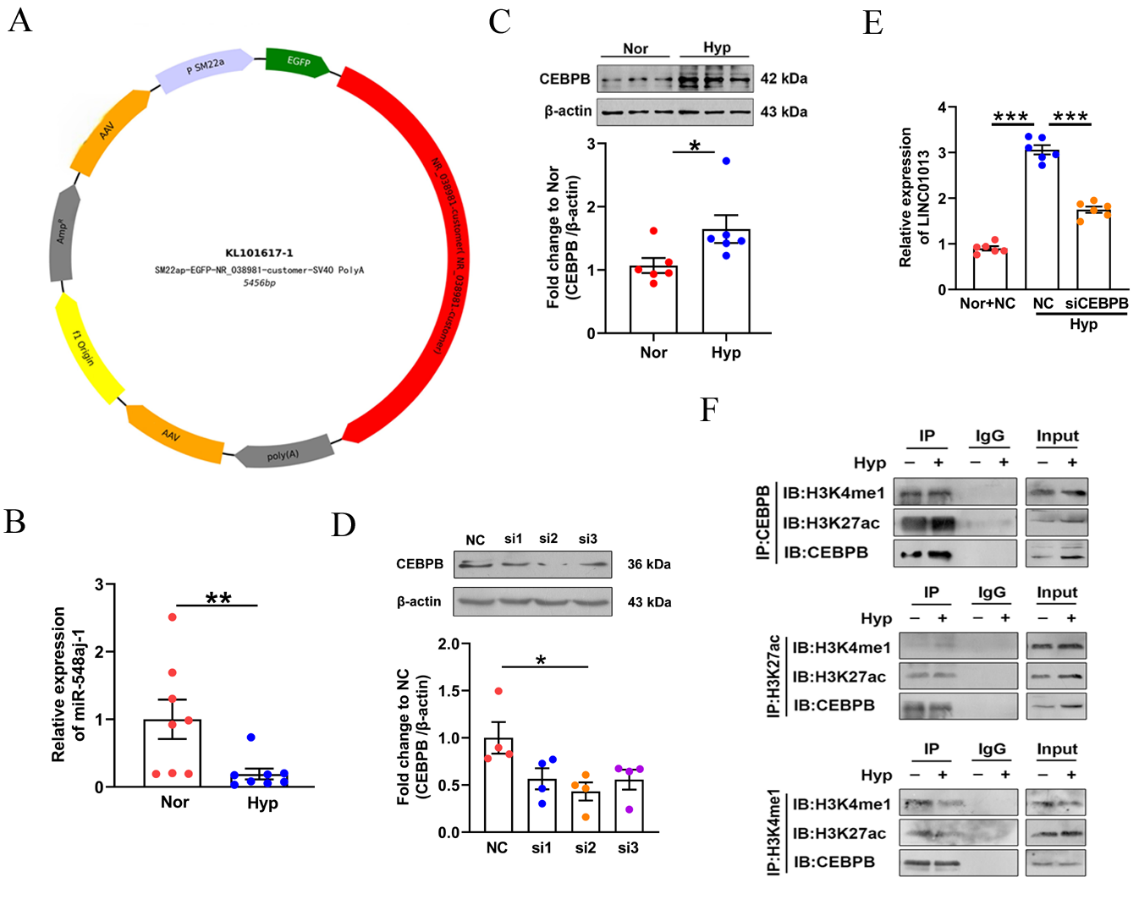


**Fig S1 A** Clonal constructs of LINC01013 targeting the smooth muscle cell-specific promoter SM22α (smooth muscle 22α) were packaged into serotype 5 adeno-associated virus (AAV5) vectors. **B** The expression levels of miR-548aj-1 in normoxic/hypoxic hPASMCs, with U6 used as a internal reference gene (n=8). **C** Western blotting analysis was used to verify the expression of CEBPB in hypoxia (n=6). **D** Interference efficiency of CEBPB siRNA were quantified by Western blotting in hPASMCs (n=4). **E** Silencing of CEBPB significantly decreased the expression of LINC01013 in hypoxic hPASMCs, with β-actin used as a internal reference gene (n=6). **F** Co-IP assay verified the interaction of CEBPB, H3K27ac, and H3K4me1 in hPASMCs. All values are presented as the mean ± SEM. Statistical analysis was performed with one-way ANOVA or Student’s t-test. **p* < 0.05, ***p* < 0.01, ****p* < 0.001. Nor, normoxia; Hyp, hypoxia; NC, negative control; si, siRNA; P SM22a, smooth muscle 22α promoter; EGFP, Enhanced Green Fluorescent Protein; AAV, Adeno-Associated Virus; F1 origin, F1 replication origin; poly (A), polyadenylate tail; Amp, Ampicillin.

**Fig S2**


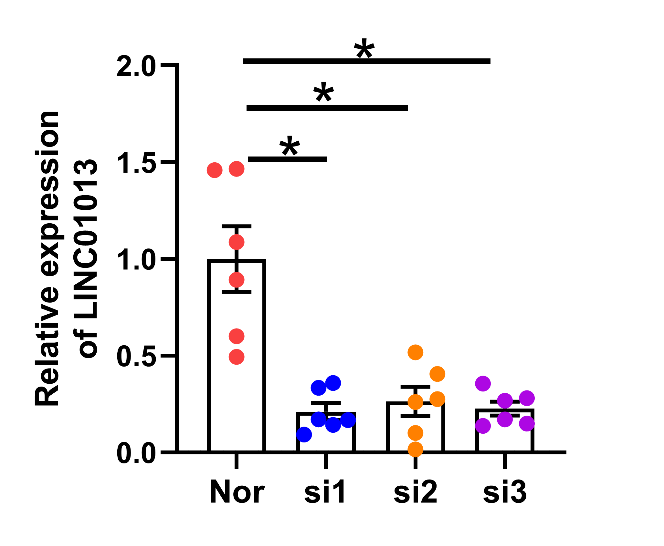


**Fig S2** Interference efficiency of LINC01013 siRNA were quantified by RT-qPCR in hPASMCs, with β-actin used as a internal reference gene (n=6). All values are presented as the mean ± SEM. Statistical analysis was performed with one-way ANOVA. **p* < 0.05. Nor, normoxia; si, siRNA.

**Fig S3**


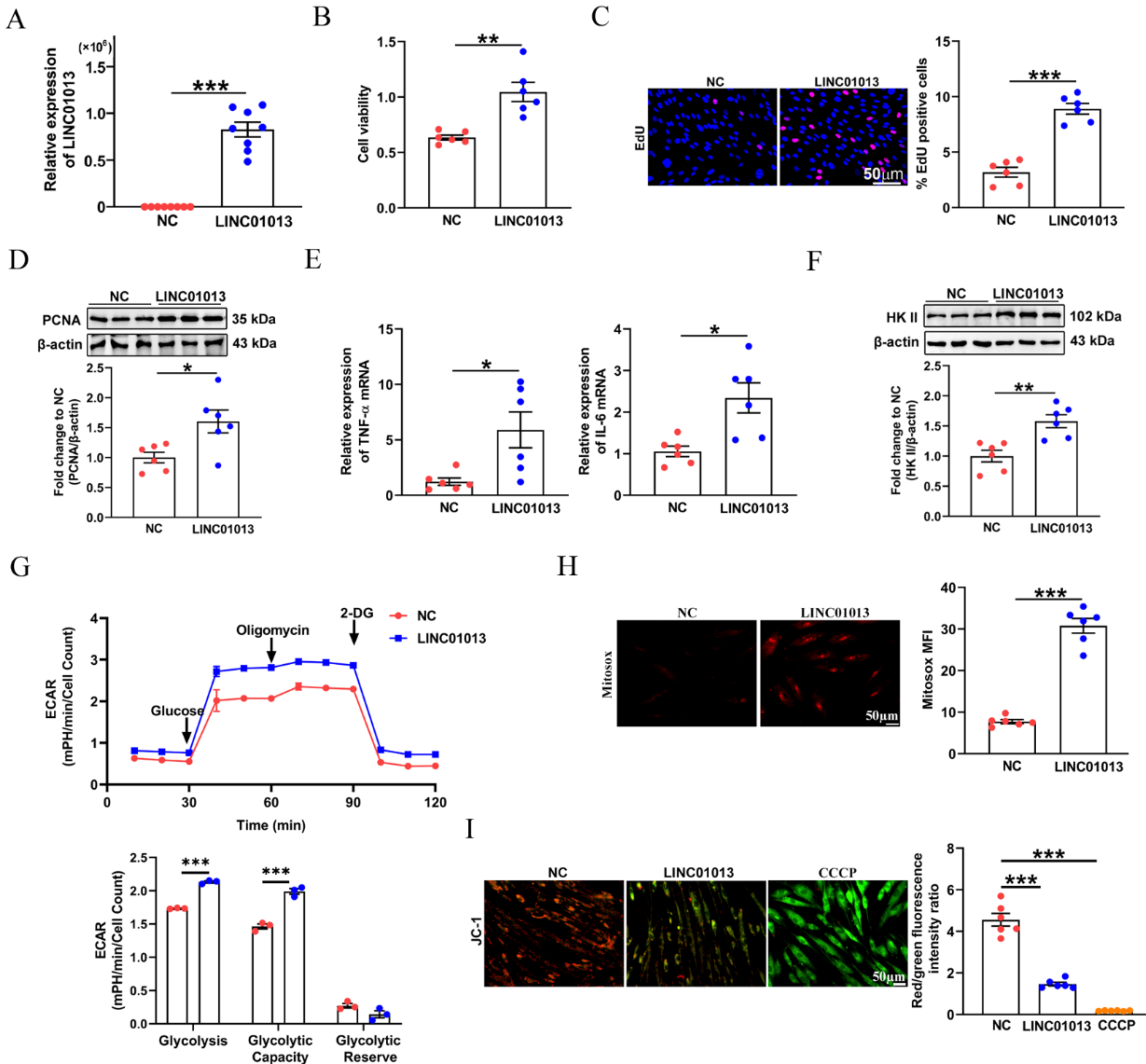


**Fig S3 A** Overexpression of LINC01013 in hPASMCs with β-actin used as a internal reference gene (n=8). **B** CCK8 analysis was performed to detect cell viability of hPASMCs after LINC01013 overexpression (n=6). **C** Representative images of hPASMCs stained with EdU (red) and DAPI (blue) (n=6). Scale bars=50 μm. **D** Expression of PCNA in hPASMCs stimulated by LINC01013 overexpression (n=6). **E** RT‒qPCR analysis showed the mRNA levels of TNF-α and IL-6 after LINC01013 overexpression, β-actin was used as a internal reference gene (n=6). **F** Western blotting analysis of HK II in hPASMCs (n=6). **G** Glycolysis and glycolytic capacity in hPASMCs following treatment with 10 mmol/L glucose and 1 µmol/L oligomycin were measured, and nonglycolytic acidification after treatment with 100 mmol/L 2-deoxyglucose (2-DG) was detected (n=3). **H** Representative images showing mitochondrial reactive oxygen species (MitoSOX, red) staining in normpxic hPASMCs (n=6). Scale bars, 50 µm. **I** Immunostaining of JC-1 aggregates (red) and monomers (green) showing changes in MMP across different groups. CCCP (10µM) was used as a positive control (n=6). Scale bars, 50 µm. All values are presented as the mean ± SEM. Statistical analysis was performed with one-way ANOVA or Student’s t-test. **p* < 0.05, ***p* < 0.01, ****p* < 0.001. NC, negative control; LINC01013, LINC01013 overexpression; MFI, Mean Fluorescence Intensity; CCCP, Carbonyl Cyanide 3-Chlorophenylhydrazone.

**Fig S4**


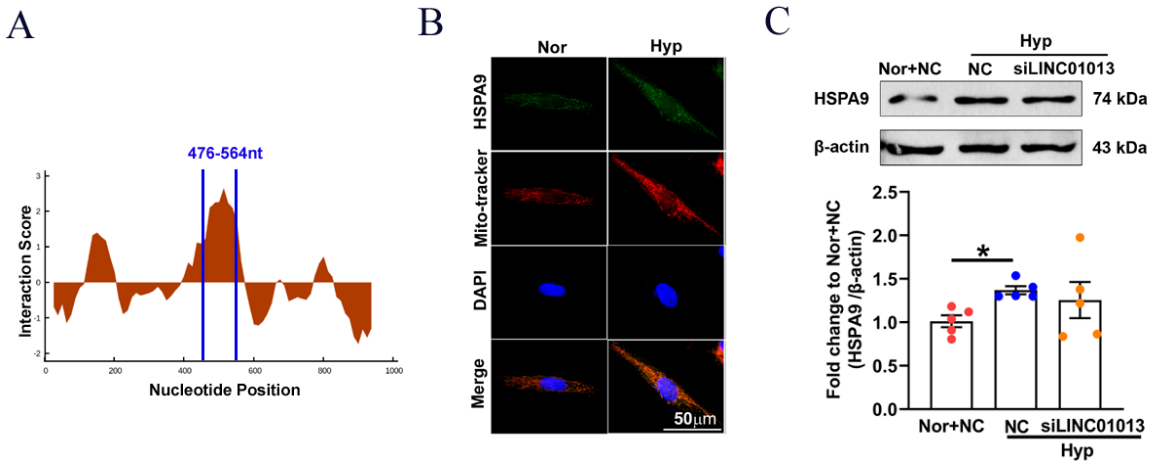


**Fig S4** **A** Interaction profile of LINC01013 and HSPA9. **B** Immunofluorescence was used to observe the colocalization of HSPA9 and mito-tracker. Scale bar, 50μm. **C** Cells were transfected with LINC01013 siRNA, and the protein expression of HSPA9 was estimated (n=5). All values are presented as the mean ± SEM. Statistical analysis was performed with one-way ANOVA. **p* < 0.05, Nor, normoxia; Hyp, hypoxia; NC, negative control; si, siRNA.

**Fig S5**


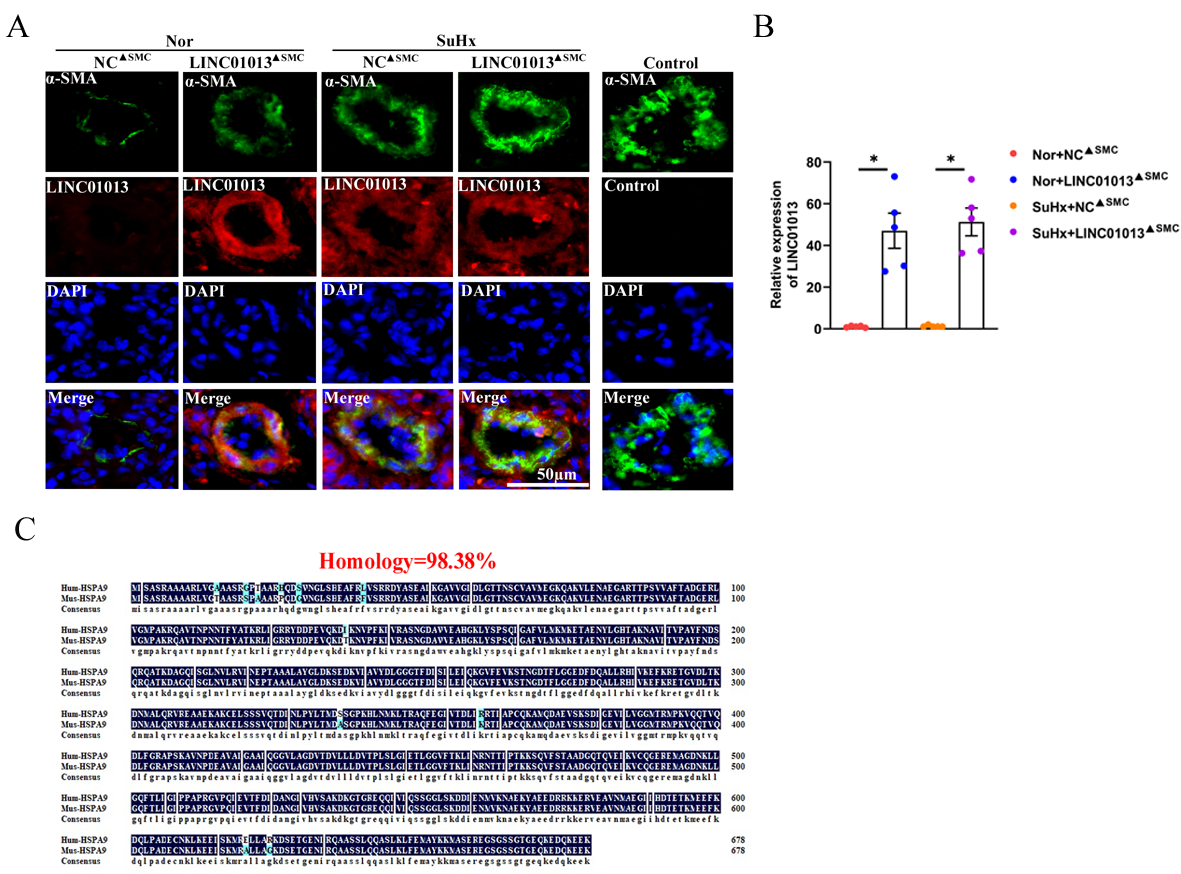


**Fig S5** **A** Representative RNA-FISH and immunofluorescence images of mouse lung sections stained with LINC01013 (red), anti-α-SMA (α-smooth muscle actin, green) antibody, and DAPI (4’,6-diamidino2-phenylindole, blue). Scale bars, 50 µm. **B** Overexpression efficiency of LINC01013 was quantified by RT–qPCR in lung tissues, with β-actin used as an internal reference gene (n=5). **C** Homology analysis of the HSPA9 between humans and mice. All values are presented as the mean ± SEM. Statistical analysis was performed with one-way ANOVA. **p* < 0.05. Nor, normoxia; SuHx, hypoxia+Su5416; NC, negative control; ▲SMC, smooth muscle cell targeting; Control, non-targeting FISH probe.

**Table S1: Prime sequences**

| **Gene Name** | **Forward primer** | **Reverse primer** |
| --- | --- | --- |
| LINC01013 | CCGGGGGCAAGGTGATTATT | CCAGACAGTGGAGAATGCGT |
| HCG20 | GGGCAGAAATGACGTGTGAG | TCTTGTGTAGCACCTGGCAC |
| LINC02709 | ACACGTTTGGCAGAGTTGGA | GGATACCAGGACTGCTGCAC |
| THSD4-AS1 | GCACATGGAGCCTTAGCTCT | CCGTGGCTTTGAGAGCATTG |
| TM4SF1-AS1 | ACTGCCAAGTGTCCGAGATG | AGGAGATTTGCCCCAGGTTG |
| LINC02225 | TGCTGCATGACTTGGGGAAT | GGCATCCGAAGGGAGACAAA |
| IL-6 | CCACCGGGAACGAAAGAGAA | GAGAAGGCAACTGGACCGAA |
| TNF-α | GGCGTGGAGCTGAGAGATAA | AGTCGGTCACCCTTCTCCAG |
| IL-1β | AGGCTGCTCTGGGATTCTCTT | TGGTGGTCGGAGATTCGTAG |
| NLRP3 | CTGGCATCTGGGGAAAACCT | GCCCTTCTGGGGAGGATAGT |
| ACTB | GCACTCTTCCAGCCTTCCTT | TGTGTTGGCGTACAGGTCTT |
| U6 | CGCTTCGGCAGCACATATAC | TTCACGAATTTGCGTGTCATC |
| 18S | CGGACACGGACAGGATTGACAG | AATCGCTCCACCAACTAAGAACGG |
| miR-548aj-1 | GCGGCGGTAAAAACTGCAATTA | ATCCAGTGCAGGGTCCGAGG |
| LINC01013(E1) | AATGCCCACATGAGGAGTGG | GTTGAGGAGCAAAACCAGCG |
| LINC01013(E2) | AGGGGCTGCAGAAGACATTC | TACTTGGGCACTGCTGGATG |
| LINC01013(E3) | CCCGCCCCATTTCACTCTTA | GGTGGTATGAAGGCAGGCAT |
| LINC01013(E4) | TTCCTTGGCTCCGAGAACTG | TGACCTGCACCTCTTGTGAC |
| LINC01013(P1) | TCACAAACCTGGACTGCTCTG | TCCTAGTCATGTGGGTGTCCT |
| LINC01013(P2) | GGAATGCAATGTAAGACCCCAG | ACTCTTCCCAAGGGGCAAAC |
| LINC01013(P3) | GTGAAAGCCCGGCTACTCTT | GCCATGTGGCAATGAATGCT |
| LINC01013(P4) | TACACATGTACCCCTTTTCCCC | AGCGTGAGGAACAACTCAAC |

**Table S2: siRNA sequences**

| **Gene Name** | **Sense（5'-3'）** | **Antisense（5'-3'）** |
| --- | --- | --- |
| siLINC01013-1 | CAGCAGUGCCCAAGUAUUATT | UAAUACUUGGGCACUGCUGTT |
| siLINC01013-2 | CACCUAAAGUUGAUACUAATT | UUAGUAUCAACUUUAGGUGTT |
| siLINC01013-3 | CAGUACAGUGGGAGAACAATT | UUGUUCUCCCACUGUACUGTT |
| NC | UUCUUCGAACGUGUCACG UTT | ACGUGACACGUUCGGAGAATT |
