# Supplementary material for: Super enhancer-driven LINC01013 mediates hypoxia-induced mitochondrial dysfunction by HSPA9 to determine pulmonary arterial smooth muscle cell fate
Source: Cell Mol Life Sci. 2026 Jan 6;83(1):57. doi: 10.1007/s00018-025-06071-3 (PMC12819934; doi:10.1007/s00018-025-06071-3)

**Uncropped blot**

Western Protein Marker I (G2086, Servicebio) was used in Western blotting.

Figure 4C


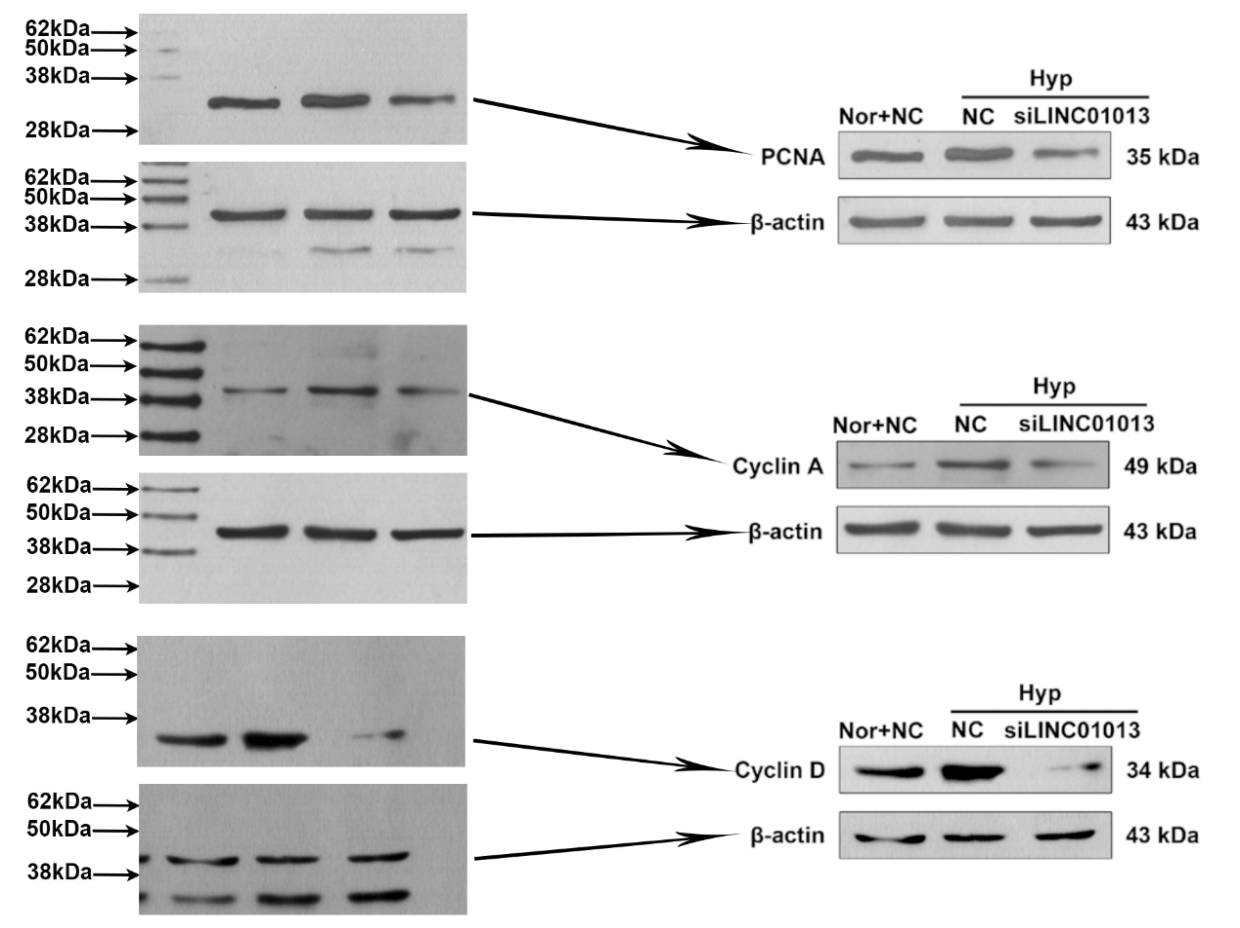


Figure 4E


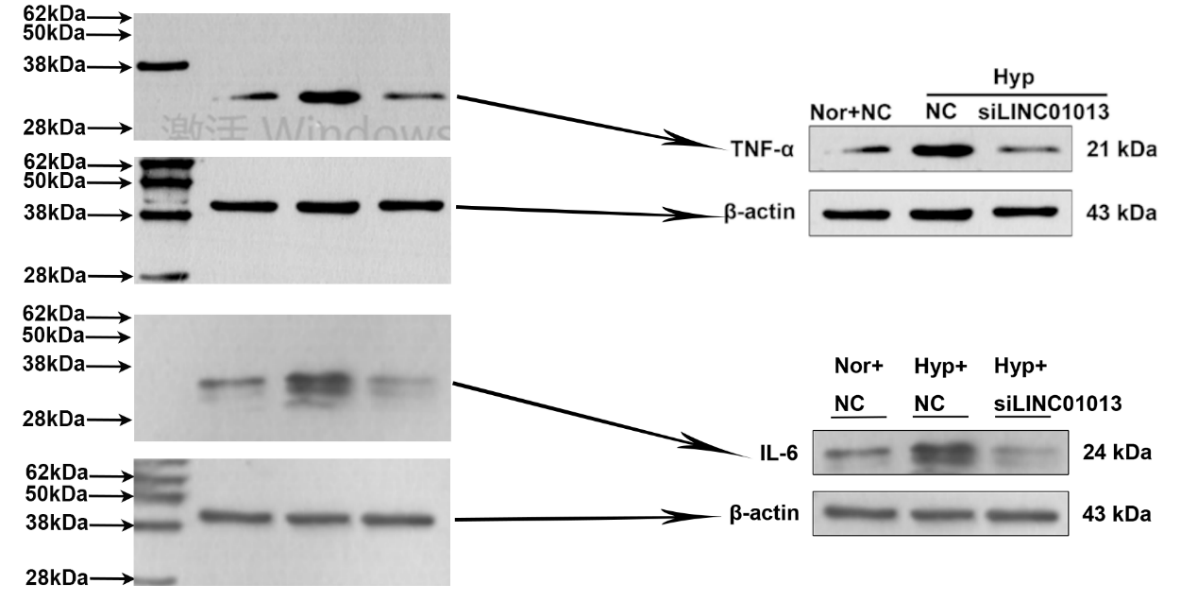


Figure 5A


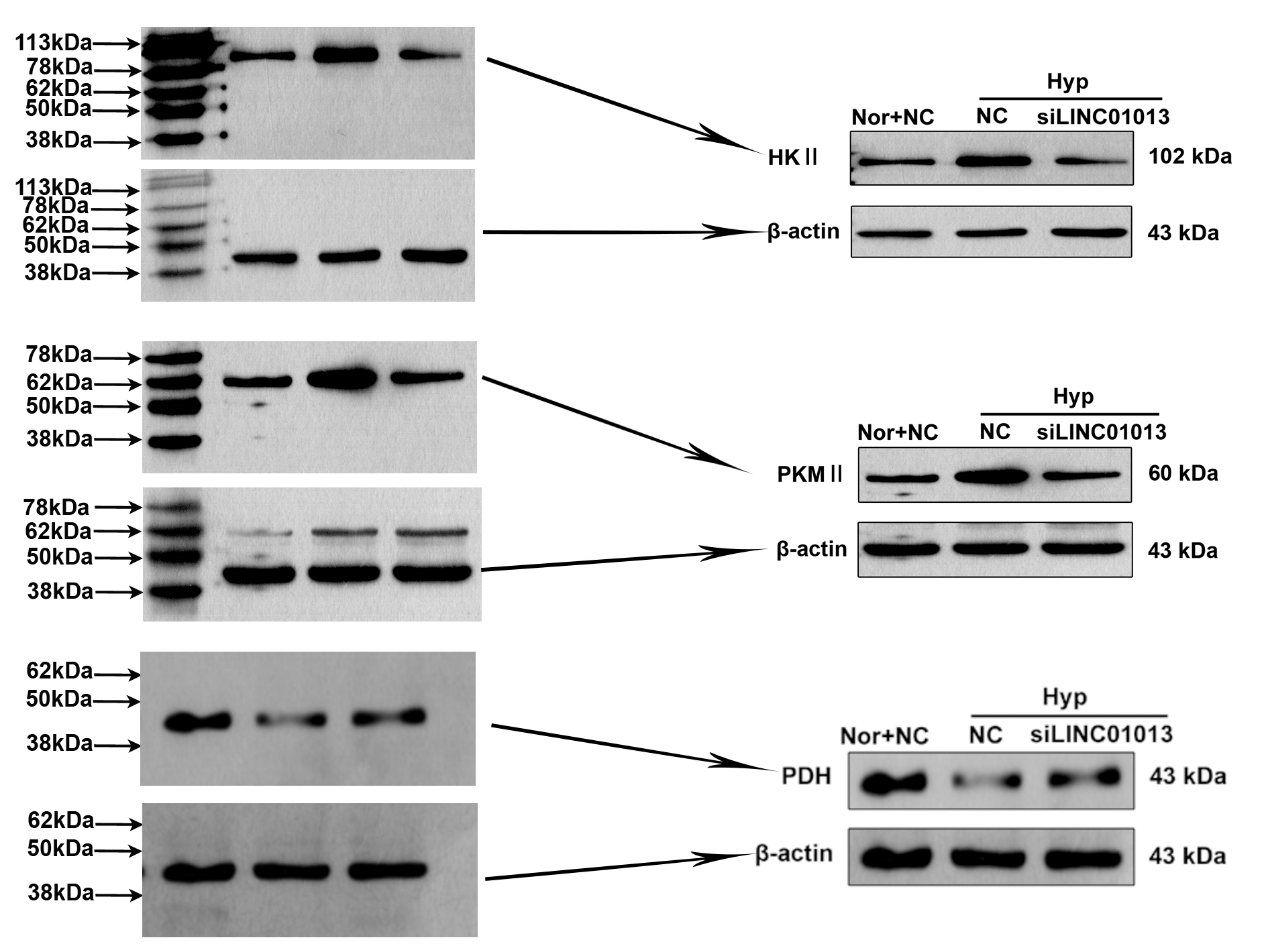


Figure 6D


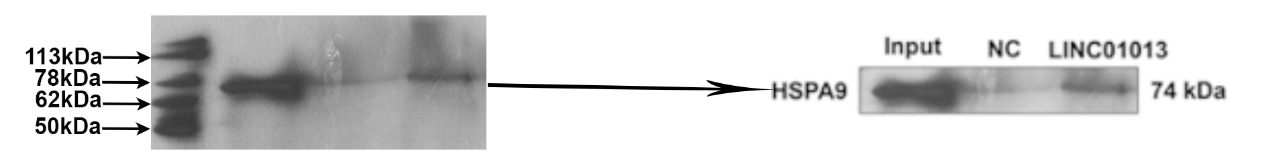


Figure 7A


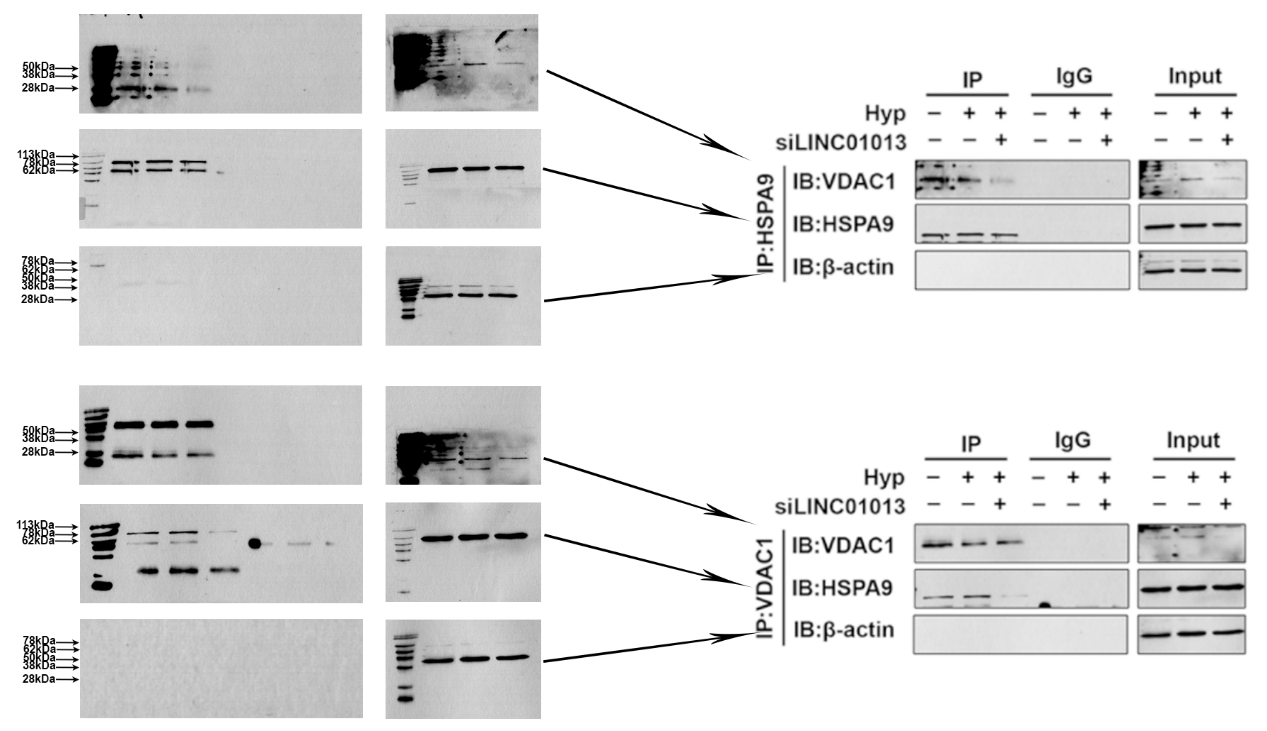


Figure 7C


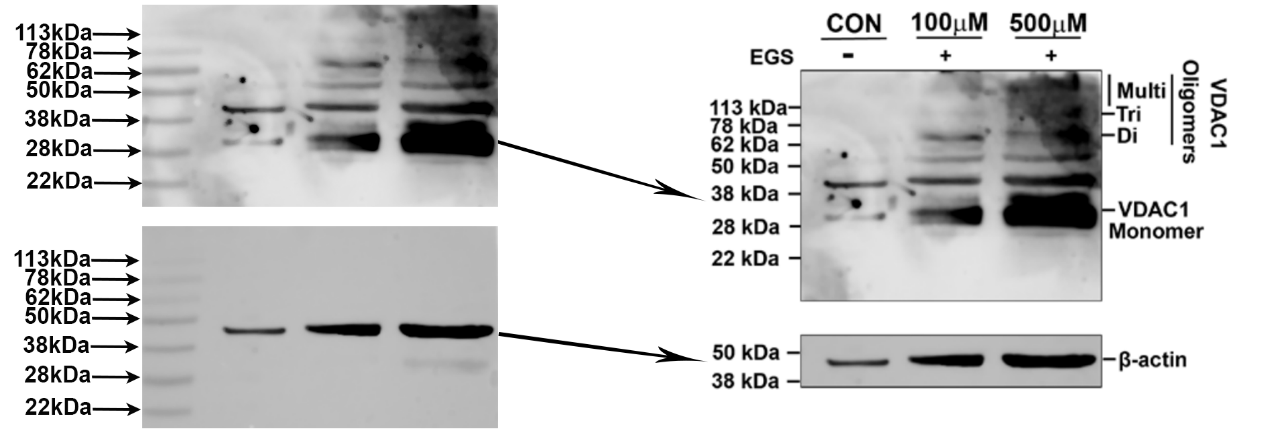


Figure 7D


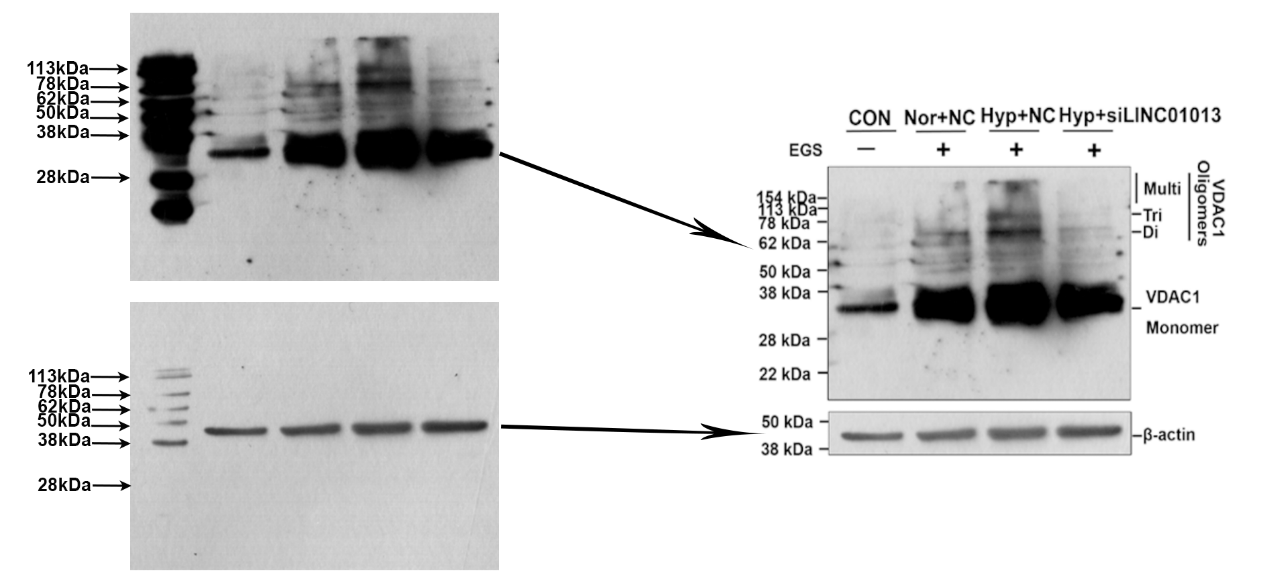


Figure 7E


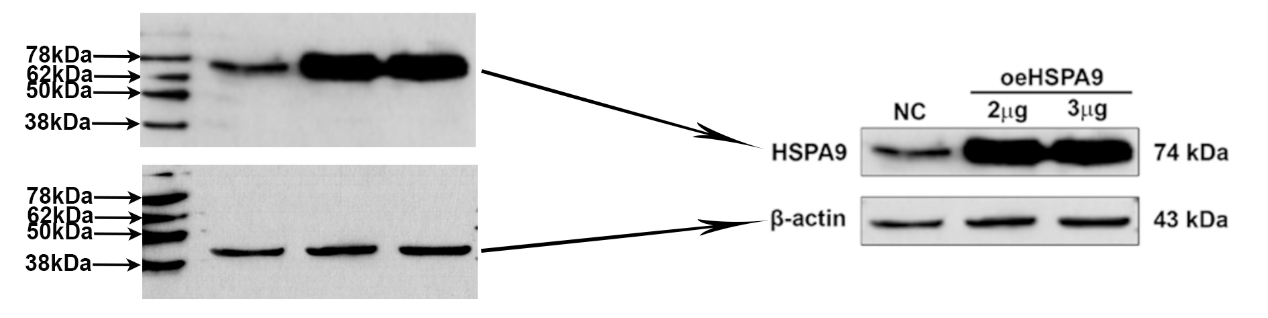


Figure 7F


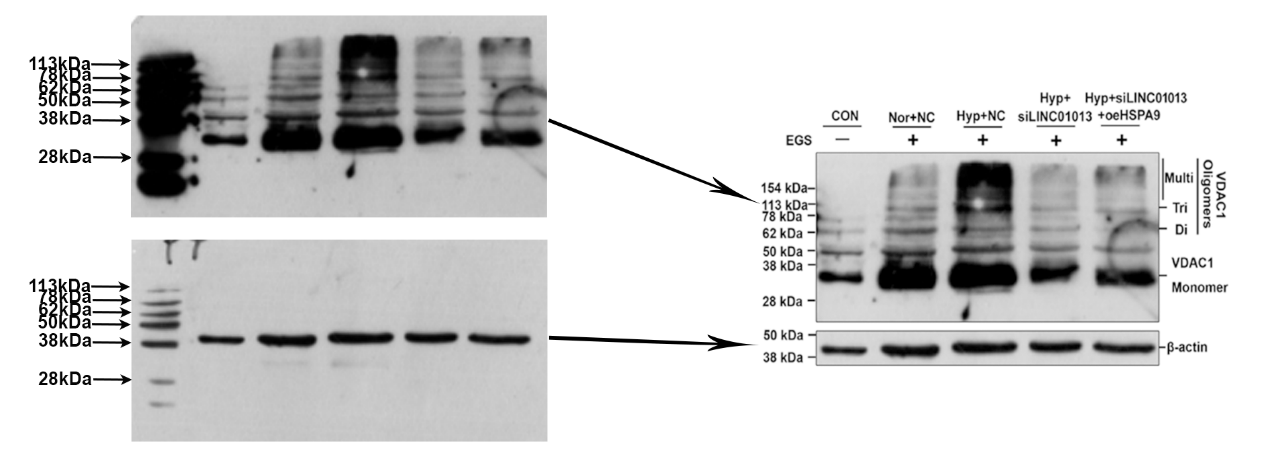


Figure 8D


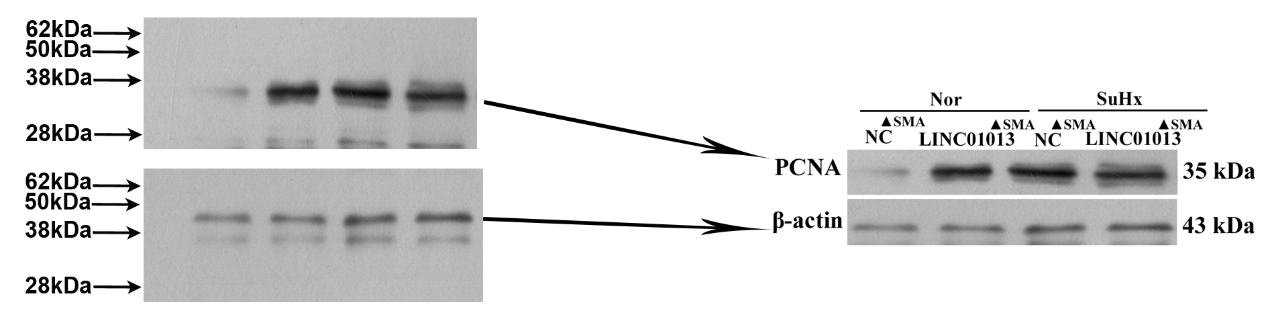


Figure 8H


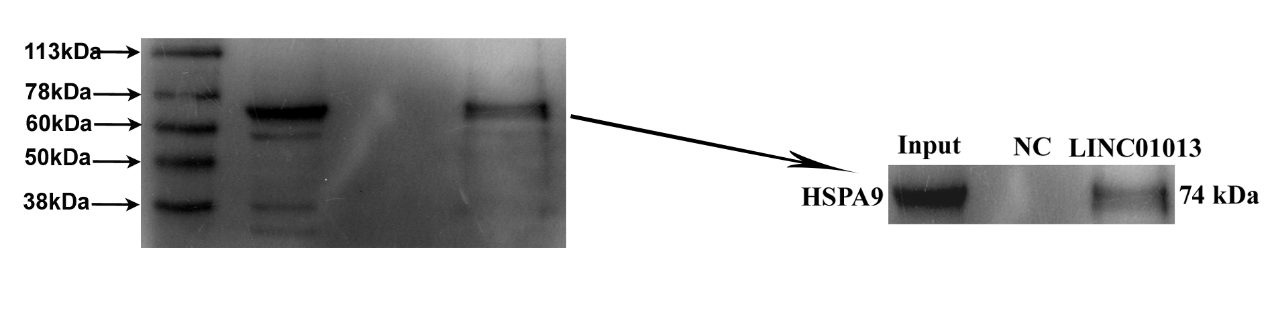


Figure S1C


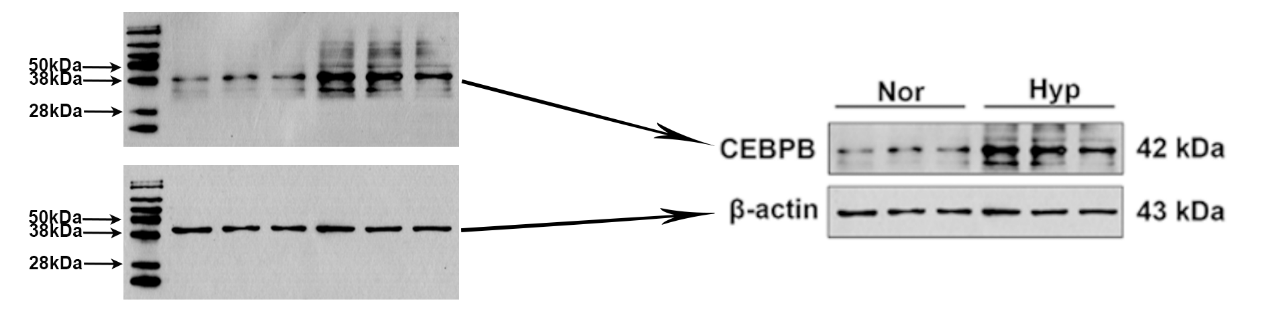


Figure S1D


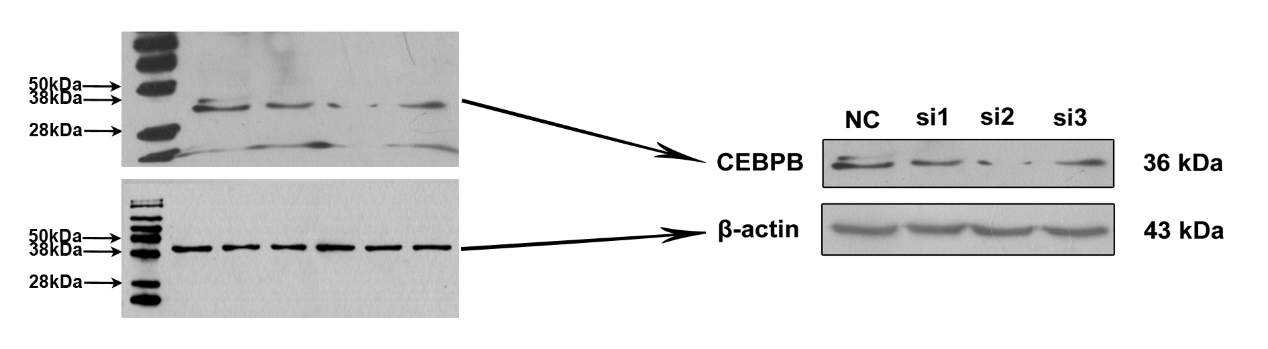


Figure S1F


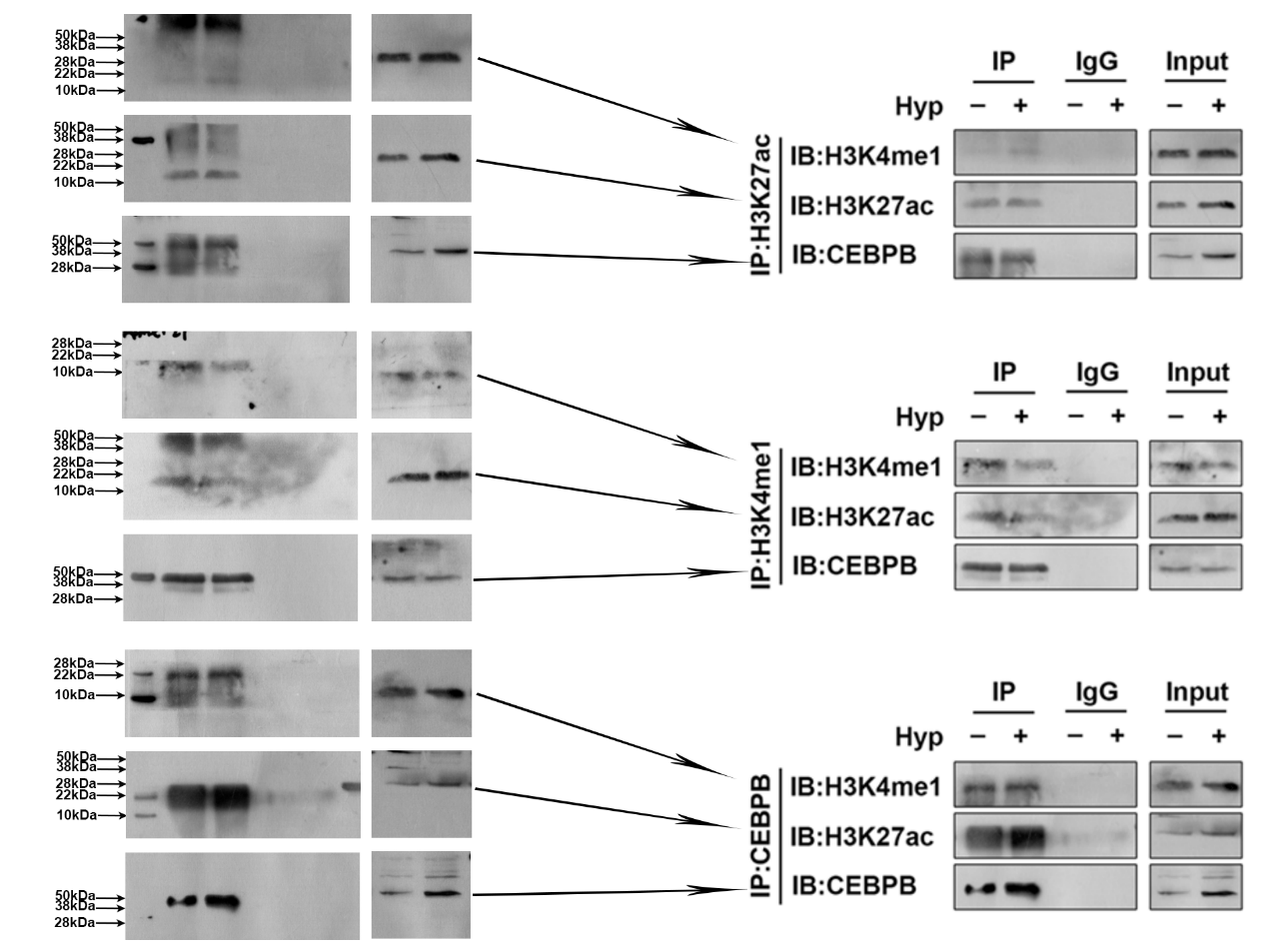


Figure S3D, S3F


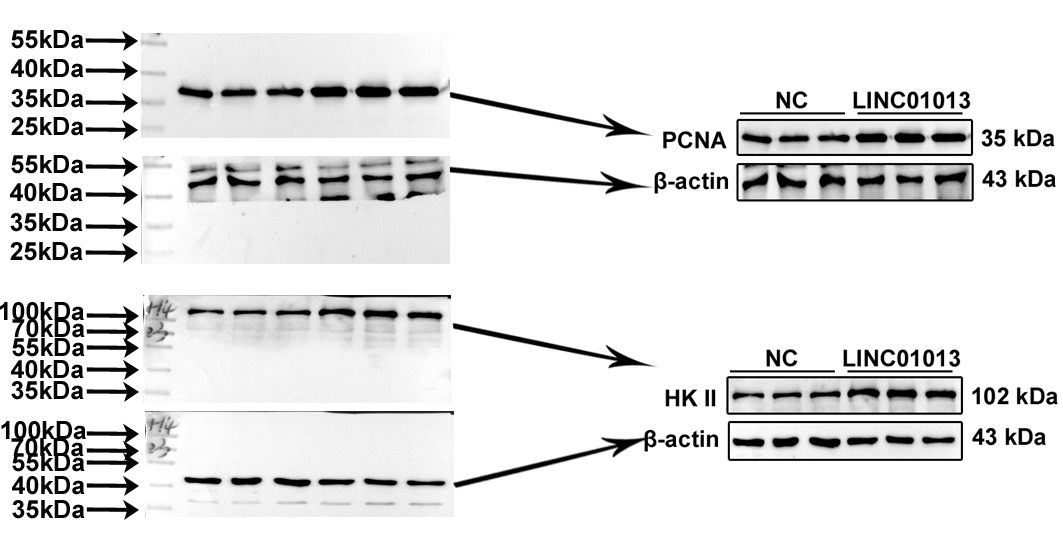


Figure S4C


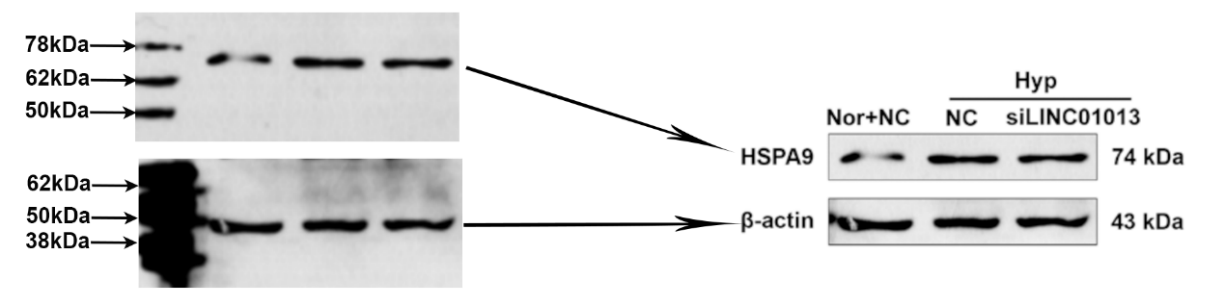

Supplement: Supplementary file 2 — Supplementary Material 2 [file 18_2025_6071_MOESM2_ESM.docx]
